# Improvement of betanin biosynthesis in *Saccharomyces cerevisiae* by metabolic engineering

**DOI:** 10.1016/j.synbio.2022.11.002

**Published:** 2022-11-12

**Authors:** Lijuan Zhang, Xue Liu, Jiawei Li, Yonghui Meng, Guang-Rong Zhao

**Affiliations:** a Frontiers Science Center for Synthetic Biology and Key Laboratory of Systems Bioengineering (Ministry of Education), School of Chemical Engineering and Technology, Tianjin University, Yaguan Road 135, Jinnan District, Tianjin, 300350, China; b Georgia Tech Shenzhen Institute, Tianjin University, Dashi 1st Rd, Nanshan District, Shenzhen, 518055, China

**Keywords:** Natural pigment, Betanin, *Saccharomyces cerevisiae*, Synthetic biology, Metabolic engineering

## Abstract

Betanin is a member of natural pigment betacyanins family and has extensive application in the food industry as an important natural red food colorant. Its relatively inefficient production in nature however hampers access to this phytochemicals through traditional crop-based manufacturing. Microbial bioproduction therefore represents an attractive alternative. Here, we present the construction of a *Saccharomyces cerevisiae* strain for betanin production. Through minimizing metabolic crosstalk, screening and modifying biosynthetic enzymes, enhancing pathway flux and optimizing fermentation conditions, a final titer of betanin of 28.7 mg/L was achieved from glucose at 25 °C in baffled shake-flask, which is the highest reported titer produced by yeast to our knowledge. This work provides a promising step towards developing synthetic yeast cell factories for *de novo* biosynthesis of value-added betanin and other betacyanins.

## 1 Introduction

Betanin is a water-soluble and red-violet natural pigment found exclusively in plants of the angiosperm order Caryophyllales and some genera of higher fungi [1,2]. Nowadays, betanin is mainly obtained from red beetroot and sold in the form of “beetroot extract” [3], which is approved as natural red colorant for use in a variety of frozen or refrigerated foods, cosmetics, and pharmaceuticals by China, European Union, and USA [1,4]. In addition to its striking colors and safety, betanin also has strong antioxidant activity [5] that can extend the shelf-life of dairy and meat products [6]. However, red beetroot has several drawbacks as the commercially used source for production of betanin: it carries adverse earthy flavors due to the presence of geosmin and various pyrazines [7,8], and the seasonal nature of planting beetroot is not ideal. Evidently, it is of interest to develop alternative sources for betanin production via synthetic biology and microbial metabolic engineering [3].

**Fig. 1:**
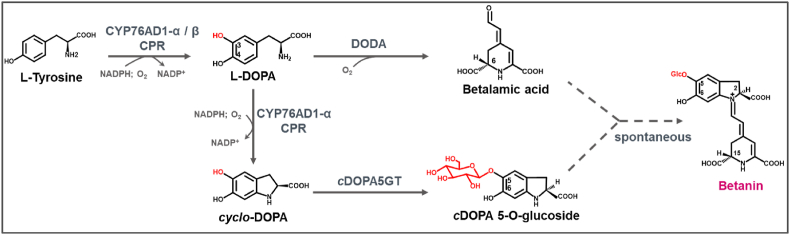
Diagram of the betanin biosynthetic pathway. Solid arrows denote enzymatic reactions and dashed arrows indicate spontaneous reactions. CYP76AD1-α/β: cytochrome P450 CYP76AD1-α or CYP76AD1-β clade proteins, CPR: cytochrome P450 reductase, DODA: DOPA-4,5-dioxygenase, *c*DOPA5GT: *cyclo*-DOPA 5-O-glucosyltransferase.

Betanin is derived from l-tyrosine via the shikimate pathway [9]. As illustrated in Fig. 1, l-tyrosine is initially 3-hydroxylated to form 3,4-dihydroxy-l-phenylalanine (l-DOPA), and l-DOPA is subsequently oxidized and cyclized to *cyclo*-DOPA. The CYP76AD1 subfamily of cytochrome P450 enzymes (P450s) takes part in this conversion process. Alternatively, l-DOPA is converted to betalamic acid by DOPA-4,5-dioxygenase enzyme (DODA). And the *cyclo*-DOPA 5-O-glucosyltransferase (*c*DOPA5GT) catalyzes glycosylation at the 5-O position of *cyclo*-DOPA [10], allowing subsequent spontaneous condensation of the *cyclo*-DOPA-5-O-glucoside with betalamic acid to form betanin.

Although the betanin biosynthetic pathway has been well studied in planta, researches involving the production of betanin in microbes has progressed very slowly, lagging far behind other tyrosine-derived compounds. Of three clades CYP76AD1 reported [11], the CYP76AD1-α and CYP76AD1-β clades are involved in the betanin biosynthesis. The CYP76AD1-α is shown to possess dual activity of tyrosine hydroxylase and l-DOPA oxidase, leading to the formation of l-DOPA and its subsequent conversion to *cyclo*-DOPA [[12], [13], [14]], and CYP76AD1-β clade exhibites merely tyrosine hydroxylase activity [15]. Due to the low enzymatic activity of CYP76AD1-α, biosynthesis of betanin is very limited in microbes [3]. What's more, the biosynthetic pathway of betanin involves multiple spontaneous reactions initiated by unstable intermediate, including *cyclo*-DOPA and betalamic acid, leading to severe metabolic crosstalk. Betalamic acid could spontaneously condense with any dissociative amino acids or amines to form diverse yellow-orange betaxanthins [16].

In the present study, *S. cerevisiae* is employed as a host for the reconstruction of betanin biosynthetic pathway. Multiple metabolic engineering efforts are made to drive the metabolic flux towards betanin biosynthesis. Initial biosynthetic enzymes in yeast is tested, and marginal production of betanin is shown in defined minimal medium by largely eliminating the formation of betaxanthins. The betanin biosynthetic pathway is improved by screening and modifying CYP76AD1-α clade proteins. Followed by optimizing fermentation conditions, 28.7 mg/L of betanin were produced from glucose, representing the highest titer reported to date. This work enabled future metabolic engineering of the unique family of pigments known as betacyanins by establishing a versatile biosynthetic platform in yeast.

## 2 Materials and methods

### 2.1 Strains and reagents

*S. cerevisiae* CEN.PK2–1C was used as the original strain for all engineered yeast strains. All of the strains constructed in this work were listed in Table 1. PCR primers were synthesized by GENEWIZ (Suzhou, China) and listed in Table S1. High-fidelity Phusion DNA polymerase and Rapid Taq Master Mix were purchased from Vazyme (Nanjing, China). All chemicals were purchased from Heowns Biochem (Tianjin, China) unless stated otherwise. The commercial red beetroot extract diluted in dextrin was used as betanin standard and purchased from Yuanye Bio-tech (Shanghai, China). l-tyrosine, l-DOPA and l-ascorbic acid were purchased from Macklin Biochem (Shanghai, China). HPLC grade of methanol and trifluoroacetic acid were purchased from Concord Tech (Tianjin, China).

**Table 1:** Strains used in this study.

Strains	Characteristics	Sources
CEN.PK2–1C	*MATa ura3-52 his3-Δ1 leu2-3_112 trp1-289, MAL2-8c SUC2*	In our lab
BET1	CEN.PK2–1C *1622b::*P_*HIS3*_–*HIS3*-P_*TDH3*_-*MjcDOPA5GT*-T_*ENO2*_-P_*HXK1*_-*MjDODA*-T_*GPM1*_	This study
BET2	BET1 *308a::*P_*TRP1*_-*TRP1*-P_*TDH3*_-*BvCYP76AD1*^*W13L*^-T_*GPD*_-P_*CCW12*_-*AtR1*-T_*ADH1*_	This study
BET3	BET1 *308a::*P_*TRP1*_-*TRP1*-P_*TDH3*_-*CqCYP76AD1*-T_*GPD*_-P_*CCW12*_-*AtR1*-T_*ADH1*_	This study
BET4	BET1 *308a::*P_*TRP1*_-*TRP1*-P_*TDH3*_-*CbCYP76AD12*-T_*GPD*_-P_*CCW12*_-*AtR1*-T_*ADH1*_	This study
BET5	BET1 *308a::*P_*TRP1*_-*TRP1*-P_*TDH3*_-*CcCYP76AD4*-T_*GPD*_-P_*CCW12*_-*AtR1*-T_*ADH1*_	This study
BET6	BET1 *308a::*P_*TRP1*_-*TRP1*-P_*TDH3*_-*HuCYP76AD1*-*1*-T_*GPD*_-P_*CCW12*_-*AtR1*-T_*ADH1*_	This study
BET7	BET1 *308a::*P_*TRP1*_-*TRP1*-P_*TDH3*_-*AcCYP76AD1*-T_*GPD*_-P_*CCW12*_-*AtR1*-T_*ADH1*_	This study
BET8	BET1 *308a::*P_*TRP1*_-*TRP1*-P_*TDH3*_-*PaCYP76AD11*-T_*GPD*_-P_*CCW12*_-*AtR1*-T_*ADH1*_	This study
BET9	BET1 *308a::*P_*TRP1*_-*TRP1*-P_*TDH3*_-*BaCYP76AD14*-T_*GPD*_-P_*CCW12*_-*AtR1*-T_*ADH1*_	This study
BET11	BET1 *308a::*P_*TRP1*_-*TRP1*-P_*TDH3*_-*CctCYP76AD4*-T_*GPD*_-P_*CCW12*_-*AtR1*-T_*ADH1*_	This study
BET12	BET1 *308a::*P_*TRP1*_-*TRP1*-P_*TDH3*_-*CctCYP76AD4*-T_*GPD*_-P_*CCW12*_-*AttR1*-T_*ADH1*_	This study
BET13	BET1 *308a::*P_*TRP1*_-*TRP1*-P_*TDH3*_-*CcCYP76AD4*-GSTSSGSSG-*AtR1*-T_*ADH1*_	This study
BET14	BET1 *308a::*P_*TRP1*_-*TRP1*-P_*TDH3*_-*CctCYP76AD4*-GSTSSGSSG-*AtR1*-T_*ADH1*_	This study
BET15	BET1 *308a::*P_*TRP1*_-*TRP1*-P_*TDH3*_-*CctCYP76AD4*-GSTSSGSSG-*AttR1*-T_*ADH1*_	This study
BET18	BET1 *308a::*P_*TRP1*_-*TRP1*-P_*TDH3*_-*CcCYP76AD4*^*W13L*^-T_*GPD*_-P_*CCW12*_-*AtR1*-T_*ADH1*_	This study
BET22	BET18 *delta::*P_*URA3*_-*URA3*-P_*TDH3*_-*CcCYP76AD4*^*W13L*^-T_*GPD*_-P_*CCW12*_-*MjcDOPA5GT*-T_*ENO2*_	This study

### 2.2 Yeast strains construction

All codon-optimized heterologous genes used in this study were synthesized by Universe Gene Technology (Tianjin, China) and listed in Table S2. High-fidelity Phusion DNA polymerase was utilized throughout the entire molecular cloning procedure. All native promoters and terminators were amplified from the *S. cerevisiae* genomic DNA by PCR. All biosynthetic genes were expressed under the control of three commonly used strong constitutive promoters, among which the *TDH3* promoter drives the expression of *MjcDOPA5GT* and *CYP76AD1-α*, the *HXK1* promoter drives the expression of *MjDODA*, and the *CCW12* promoter drives the expression of *AtR1*. Moreover, the biosynthetic gene expression cassettes were chromosomally integrated. Functional expression cassettes of heterologous genes were assembled according to overlap-extension PCR (OE-PCR) procedure, and transformed into *S. cerevisiae* competent cells by the LiAc method, and finally integrated at selected genomic loci via the homologous recombination method [17]. All used homology sequences of chromosomal loci were listed in Table S3.

### 2.3 Media and cultivation conditions

Yeast strains for preparation of competent cells were cultivated in yeast extract peptone dextrose (YPD) medium (20 g/L glucose, 20 g/L tryptone, and 10 g/L yeast extract). Strains containing auxotrophic markers (TRP1, URA3 or LEU2) were selected on synthetic complete (SC) medium (20 g/L glucose, 6.7 g/L yeast nitrogen base without amino acids, and 2 g/L amino acid mixed powder) lacking appropriate amino acids.

Shake-flask fermentations for the production of betanin were performed in defined minimal (DM) medium (20 g/L glucose, 7.6 g/L yeast nitrogen base without amino acids, 2 g/L inositol, 400 mg/L calcium pantothenate, 400 mg/L pyridoxin HCl, 400 mg/L thiamine HCl, 2 mg/L biotin) lacking appropriate amino acids [13]. Except where noted, 10 mM l-ascorbic acid was added in DM medium as a reducing agent to prevent *cyclo*-DOPA oxidation and polymerization to form melanin, and 500 mg/L l-DOPA or l-tyrosine was supplemented as the substrate when necessary. Three independent single colonies, with the relevant genetic modifications, were inoculated into 15 mL test tubes with 3 mL of SC medium, and incubated at 30 °C with 250 rpm agitation overnight. Precultures were then diluted into 5 mL of fresh SC medium in test tubes, and cultivated overnight at 30 °C, 250 rpm. Subsequently, the seed cultures were transferred into a 250 mL shake-flasks carrying 50 mL of DM medium with an initial optical density measured at 600 nm of 0.1 and cultivated at 30 °C, 250 rpm for 72 h.

### 2.4 Biomass and metabolite analysis

The optical density of yeast cells was measured at 600 nm (OD_600_) by TU-1810 spectrophotometer. Whereafter, the fermentation broth was centrifuged at 12,000 rpm for 10 min, and supernatant was used for analysis of metabolites. The absorbance spectra of culture supernatants were obtained on the Thermo Scientific™ Varioskan™ LUX with a 1 nm wavelength step. For the detection of betanin and metabolic intermediates l-tyrosine, l-DOPA and betalamic acid, 10 μL sample was filtered through 0.22 μm pore-sized syringe filter, and analyzed on HITACHI HPLC system equipped with a C18 column (250 × 4.6 mm with a particle size of 5 μm) connected to a UV detector (HITACHI, Japan). The column was kept at 25 °C, and a flow rate set to 1 mL/min was used. Betanin was measured with a mobile phase of 20% methanol, 80% water, and 0.1% trifluoroacetic acid at 535 nm. l-tyrosine and l-DOPA were determined at 280 nm with the mobile phase of 5% acetonitrile, 95% water, and 0.1% trifluoroacetic acid. Betalamic acid was detected at 405 nm with gradient program which was started with 95% solvent A (1.5% phosphoric acid in water) and 5% solvent B (methanol), changed linearly to 60% solvent A and 40% solvent B in 20 min, then returned to 95% solvent A and 5% solvent B in 1 min, and finally remained for 4 min [16]. The concentrations of residual glucose and ethanol were determined by HPLC analysis. Briefly, 10 μL filtered sample was analyzed on HITACHI HPLC system equipped with an Aminex HPX-87H column (Bio-Rad) connected to a refractive index detector (Waters 2414, Milford, USA). The column was eluted with 5 mM H_2_SO_4_ at a flow rate of 0.6 mL/min at 65 °C for 25 min. Betanin, l-tyrosine, l-DOPA, glucose, and ethanol were quantified using a five-point calibration curve and the *R*^*2*^ coefficient for the calibration curve was higher than 0.99. Since betalamic acid standard is not available, the amount of betalamic acid was indicated by the peak area of HPLC analysis.

Since pure betanin molecule is not commercially available, the quantification of betanin in this study was selectively referred to the method of Grewal et al. [3]. Using the Beer-Lambert law with betanin molar absorption coefficient (*ε*) of 65,000 M^−1^ cm^−1^ at 535 nm [18] and betanin molecular weight of 550.48 g/mol, the amount of molecular betanin in the commercial red beetroot extract was determined by ultraviolet–visible spectrophotometer. Betanin was identified by LC-MS analysis using a Synapt G2-Si Q-TOF mass spectrometer coupled with an ACQUITY UPLC system (Waters, USA) run in positive electrospray (ESI^+^) mode. We generated a HPLC spectral calibration curve of betanin based on serial dilutions of the commercial red beetroot extract at 535 nm. Under the same conditions, betanin in fermentation broth was analyzed by HPLC, and the concentration was calculated by betanin calibration curve.

## 3 Results and discussion

### 3.1 Screening of starting medium for betanin biosynthesis of engineered *S. cerevisiae*

To develop the plasmid-free *S. cerevisiae* strain for producing betanin, we introduced the genes encoding MjDODA and Mj*c*DOPA5GT into the *1622b* locus of CEN.PK2–1C chromosome [19] to generate strain BET1. Then, we integrated the mutant BvCYP76AD1^W13L^ from *Beta vulgaris* [13] and cytochrome P450 reductase from *Arabidopsis thaliana* (AtR1) [12] at the *308a* locus [19] of strain BET1 to generate strain BET2. During the biosynthesis of betanin in yeast, betaxanthins would be produced as byproducts [3], due to metabolic crosstalk initiated by the spontaneous reactions of betalamic acid with amino acids and amines in the medium. Generally, the SC and YPD media are commonly used in yeast metabolic engineering. Our study showed that SC medium was superior to YPD medium for yeast producing genistein, a compound derived from tyrosine [26]. Although the original DM medium lacked amino acids or amines, additional nutritional amino acids (histidine and methionine) were added to support the growth of auxotrophic yeast when it was used to produce betanin [3] and betanidin [13]. In our previous study on *de novo* production of yellow betaxanthins in *Escherichia coli*, histidine, lysine, and arginine with the positively charged side chains were found to have higher conjugation affinities toward betalamic acid [16]. Accordingly, we first tested the effects of three starting media on betanin production in this work.

**Fig. 2:**
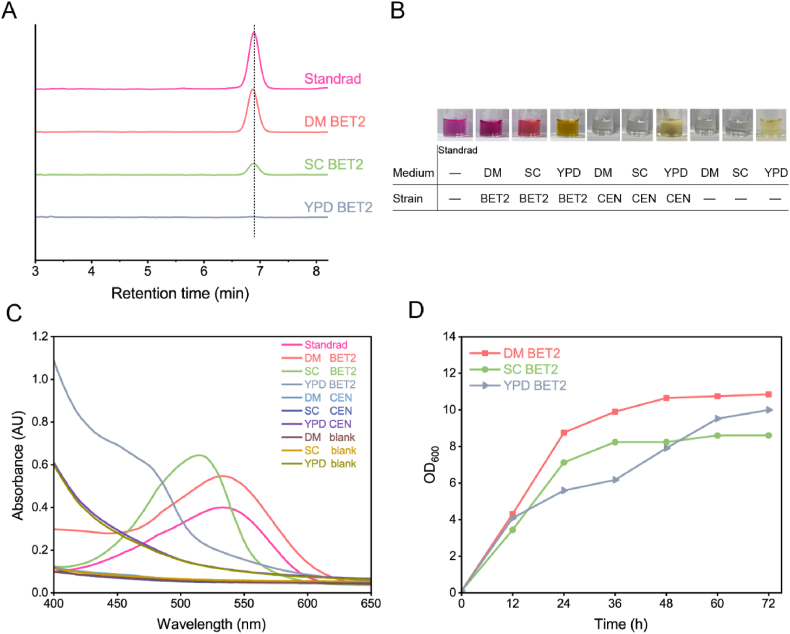
Biosynthesis of betanin from l-tyrosine by engineered *S. cerevisiae* BET2 in different media. (A) HPLC spectra of the culture supernatant of strain BET2. (B) Culture supernatant colors. (C) Absorbance spectra of the culture supernatants. (D) Growth profiles of strain BET2 for 72 h. CEN indicates strain CEN.PK2–1C and blank (−) indicates liquid medium without yeast strain.

Strain BET2 was inoculated into liquid DM, SC, and YPD media supplemented with 500 mg/L l-tyrosine, respectively. After 72 h of fermentation in DM and SC media, strain BET2 produced a new peak in HPLC spectra, which had the same retention time with betanin standard (Fig. 2A), while no peak was detected in YPD medium. The compound corresponding to the peak was subsequently identified to be betanin by high resolution LC-MS analysis, which displayed a molecular ion at *m/z* 551.1485 ([M+H]^+^) (Fig. S1). Notably, the culture supernatant colors were varied when different media were used. The red-violet color was shown in DM medium, and the tomato color was observed in SC medium, whereas the yellow color was presented in YPD medium (Fig. 2B). Subsequently, absorbance scan of these supernatants was carried out. The results showed that absorbance curve of the culture supernatant from DM medium was the same as that of betanin standard, with the maximum absorbance at 535 nm (Fig. 2C). The culture supernatant from SC medium exhibited a strong absorbance between 470 nm and 530 nm, within the betanin and betaxanthins superimposed absorption range, and its maximum absorption wavelength was 515 nm, indicating that it was a mixture of red-violet betanin and yellow betaxanthins (Fig. 2C). It might be that more than ten amino acids and amines in SC medium competed the production of betanin. While the absorbance of culture supernatant from YPD medium was similar with yellow betaxanthins as previous reports [13,20], indicating that the biosynthesis of betanin was interrupted by high amounts of amino acids in YPD medium. As shown in Fig. 2C, the absorbance peak at 460–480 nm was not observed in culture supernatant from DM medium, indicating that precursor l-tyrosine and nutrient uracil and leucine might be less condense with betalamic acid to form betaxanthins, consistent with the previous study [16]. Thus, the less amino acids in DM medium might reduce the formation of byproduct betaxanthins and improve the biosynthesis of betanin. In addition to excellent performance of DM medium in terms of reducing metabolic crosstalk, as shown in Fig. 2D, strain BET2 grew better in DM medium than in SC or YPD medium, indicating a significant biomass advantage. We speculated that more vitamins in DM medium might be beneficial to yeast growth. Taking together, DM medium is more suitable than SC and YPD media for betanin production in *S. cerevisiae* and used for the following studies.

### 3.2 Screening of *cyclo*-DOPA synthase for improvement of betanin production

**Fig. 3:**
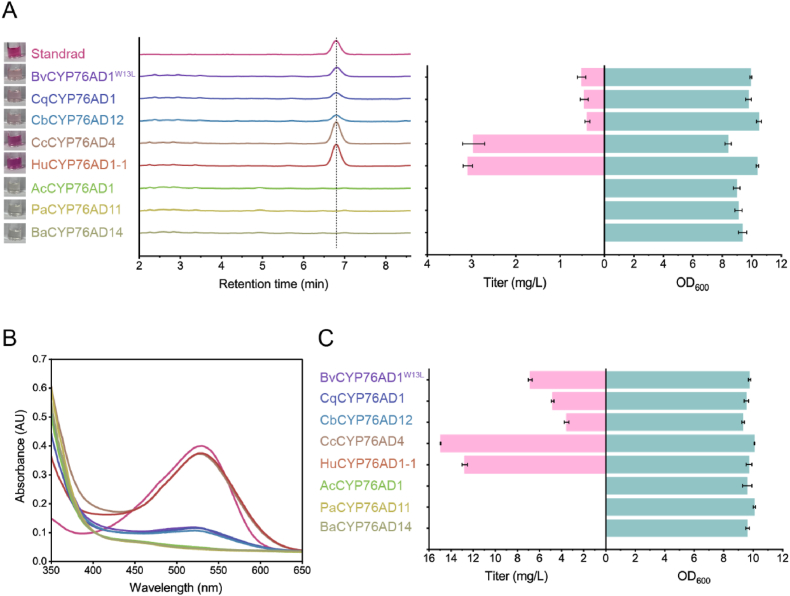
Performance of different CYP76AD1-α clade proteins in betanin biosynthesis. (A) HPLC spectra and betanin production from l-tyrosine by strains expressing different CYP76AD1-α clade proteins. (B) Absorbance spectra of the culture supernatants in A. (C) Betanin production from l-DOPA by strains expressing different CYP76AD1-α clade proteins. Error bars represent means ± S.D. of three biological replicates.

To improve the production of betanin, we performed the screen of *cyclo*-DOPA synthase in strain BET1, and seven annotated CYP76AD1-α clade proteins from Caryophyllales were chosen as enzyme candidates in this work (Fig. S2). Identities of these proteins with BvCYP76AD1 ranged from 74% to 88%, and most of them were not functionally characterized, except for CcCYP76AD4 [13] (Table S4). Seven CYP76AD1-α clade proteins from different plant species were co-expressed with AtR1 in the chromosome of strain BET1, generating strains BET3 to BET9, respectively. The strain BET2 expressing gene *BvCYP76AD1*^*W13L*^ was used as positive control. After 72 h of shake-flask fermentation in DM medium supplemented with l-tyrosine, as shown in Fig. 3A, four of seven strains made the red-violet color culture supernatants that were visible to the naked eye, whereas the rest three strains expressing AcCYP76AD1, PaCYP76AD11, and BaCYP76AD14 did not. Subsequent analysis displayed that those red-violet colored culture supernatants produced the peaks of betanin in HPLC spectra with the maximum absorbance at 535 nm (Fig. 3B), while there was no detectable betanin in colorless supernatants. The betanin titers were quantified, and the results showed that strains expressing CqCYP76AD1 and CbCYP76AD12 synthesized small amounts of betanin (∼0.5 mg/L), comparable to strain BET2 expressing BvCYP76AD1^W13L^, while strains expressing CcCYP76AD4 and HuCYP76AD1-1 yielded 3.0 and 3.1 mg/L of betanin, respectively, approximately 6-fold of strain BET2 (Fig. 3A).

Since the CYP76AD1-α clade proteins have the bifunctional activity for both l-tyrosine and l-DOPA as substrates, we further tested the capacity of betanin synthesis from l-DOPA. Strains BET2 to BET9 were inoculated into DM medium supplemented with 500 mg/L l-DOPA. Similar to the substrate l-tyrosine, strains BET7 to BET9 did not produce betanin, indicating that AcCYP76AD1, PaCYP76AD11 and BaCYP76AD14 do not have the ability to synthesize *cyclo*-DOPA from l-DOPA. Compared to BvCYP76AD1^W13L^ for betanin biosynthesis from l-DOPA, CqCYP76AD1 and CbCYP76AD12 were less efficient, while CcCYP76AD4 and HuCYP76AD1-1 were better, 15.0 and 12.8 mg/L of betanin were obtained (Fig. 3C), respectively. Thus, CcCYP76AD4 performed best among all enzyme candidates and has the potential to become a more efficient enzyme for betanin synthesis through further engineering. These results indicated that the efficient CYP76AD1-α clade protein was crucial for improvement of betanin production in yeast.

### 3.3 Improving betanin production via biosynthetic pathway optimization

**Fig. 4:**
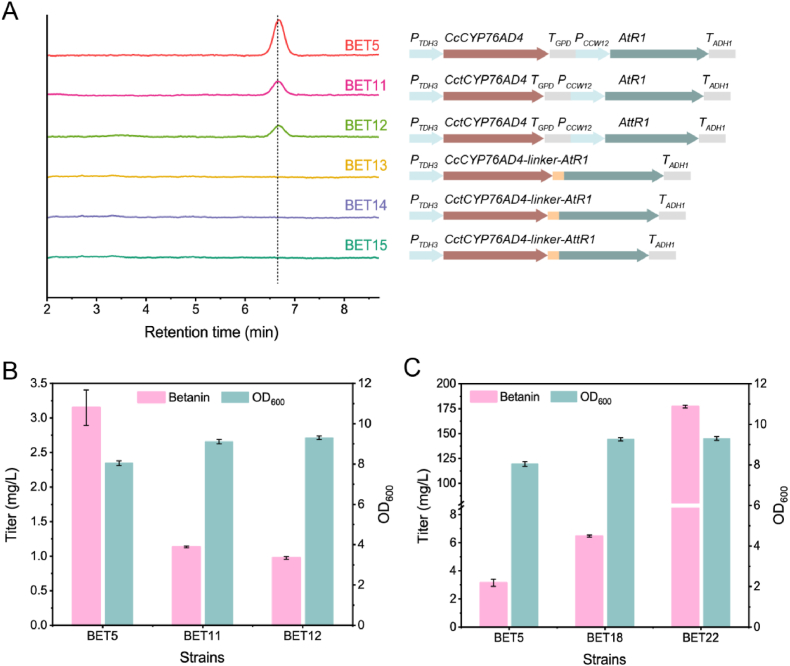
Optimization of betanin biosynthetic pathway. (A) HPLC spectra of fermentation broth of strains BET11 to BET15. (B) Betanin production from l-tyrosine by strains BET11 and BET12. (C) Betanin biosynthesis of mutant CcCYP76AD4^W13L^ in strain BET18 and chromosomal integration of the *delta* locus in strain BET22.

Truncating the N-terminal membrane anchor domain of P450s or CPRs, and fusing P450s with CPRs have been successfully employed to enhance the production of protopanaxadiol [21], levopimaric acid [22] and dihydroartemisinic acid [23] in *S. cerevisiae*. Here, these strategies were implemented to improve betanin production. The predicted transmembrane sequences, namely the N-terminal 22 amino acids of CcCYP76AD4 and 46 amino acids of AtR1 were truncated, resulting in CctCYP76AD4 and AttR1, respectively. Then two combinations of CctCYP76AD4 with AtR1 or modified AttR1 were designed, generating strains BET11 and BET12 (Fig. 4A). Unexpectedly, as shown in Fig. 4B, the fermentation results showed that small amounts of betanin (∼1.0 mg/L) were produced from l-tyrosine, a remarkable decrease in productivity compared to strain BET5, indicating that the truncation of the N-terminal transmembrane domain of CcCYP76AD4 and AtR1 was impaired for biosynthesis of betanin in yeast. Furthermore, three fusion versions of CcCYP76AD4 or CctCYP76AD4 with AtR1 or AttR1 were constructed. Disappointedly, betanin was not detected in the fermentation broth of strains BET13, BET14 and BET15 (Fig. 4B), indicating that CcCYP76AD4-AtR1 fusion with or without truncation lost the catalytic activity for synthesis of *cyclo*-DOPA. Our results indicated that N-terminal transmembrane domain and individual expression were crucial for efficient catalysis of CcCYP76AD4 in yeast, which contradicted previous reports [21,22], this discrepancy could be attributed to the difference of P450s. Thus, we made other engineering efforts to maximize catalytic efficiency of CcCYP76AD4.

Previous study showed that mutant BvCYP76AD1^W13L^ improved enzymatic activity of tyrosine hydroxylase and increased the biosynthesis of betanidin compared to wild-type in yeast [13], which aroused our interest in the single mutant of CcCYP76AD4 and its effect on betanin biosynthesis. We performed a protein alignment of BvCYP76AD1 with its seven orthologs used in this study, which showed that the W13 residue was conserved in CcCYP76AD4 (Fig. S3). Therefore, mutant CcCYP76AD4^W13L^ replaced BvCYP76AD1^W13L^ in strain BET2 to generate strain BET18. As expected, the introduction of the mutation of CcCYP76AD4^W13L^ exhibited remarkable improvement of the production, leading to 6.5 mg/L of betanin, 2.1-fold of that by the wild-type (Fig. 4C). Encouraged by this, we overexpressed the engineered version of CcCYP76AD4^W13L^ and Mj*c*DOPA5GT in the *delta* loci of strain BET18, the resulting strain BET22 increased the betanin titer to 177.2 mg/L from l-tyrosine, 26.4-fold higher than strain BET18 (Fig. 4C). The results clearly showed that intensifying the biosynthetic enzymes indeed dramatically improve the betanin titer.

### 3.4 Optimization of fermentation conditions for *De novo* biosynthesis of betanin from glucose

**Fig. 5:**
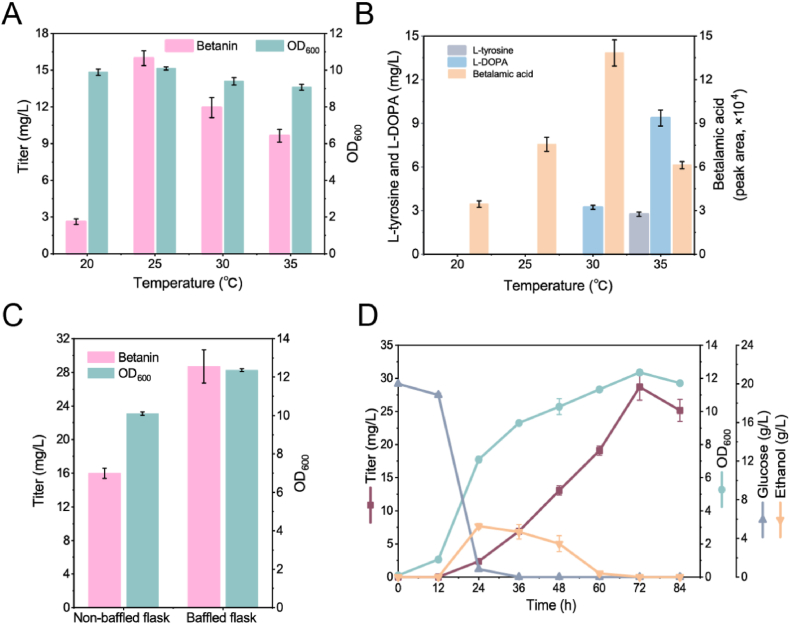
Optimization of fermentation conditions for the biosynthesis of betanin from glucose in strain BET22. (A) Effects of different fermentation temperatures on betanin biosynthesis. (B) The amounts of l-tyrosine, l-DOPA and betalamic acid at different temperatures. (C) Effect of dissolved oxygen on betanin biosynthesis. (D) Fermentation profiles of strain BET22 cultured in shake-flask with baffles for 84 h.

Upon the efficient biosynthesis of betanin from l-tyrosine, we further evaluated the ability of strain BET22 to *de novo* produce betanin using glucose as carbon source. Given the influences of fermentation temperature on cell growth and enzyme activity [[24], [25], [26]], we firstly inoculated strain BET22 in shake-flasks and performed the fermentation for 72 h at four different temperatures. The fermentation results showed that the temperature had less effect on the yeast growth, but significantly impacted on betanin production (Fig. 5A). 16.0 mg/L of betanin was obtained at the optimal temperature 25 °C, 610%, 134% and 166% of that at 20 °C, 30 °C, and 35 °C, respectively. Correspondingly, a more vivid red-violet color was observed in the culture supernatant at 25 °C (Fig. S4). Thus, cultivation temperature at 25 °C was more suitable for betanin production. Additionally, l-tyrosine and l-DOPA were not detected at 20 °C and 25 °C, while 3.2 mg/L of l-DOPA was accumulated at 30 °C, and l-DOPA (9.4 mg/L) was increased along with accumulation of l-tyrosine (2.7 mg/L) at 35 °C (Fig. 5B and Fig. S5). The amount of betalamic acid increased with increasing cultivation temperature, and highest at 30 °C with 180% of that at 25 °C (Fig. 5B). The results suggested that lower accumualtion of the metabolic intermediates might contribute the higher production of betanin at 25 °C. The production of betanin and geinistein in yeast [26] was optimal at 25 °C instead of the optimal growth temperature at 30 °C, which should be given more attetion by metabolic engineers for natural product biosynthesis.

Furthermore, the oxygen supply is an important consideration in the engineering of betanin recombinant pathway involving P450s [3]. To explore the effect of oxygen supply, we cultivated strain BET22 in non-baffled and baffled shake-flasks at 25 °C, and the results were shown in Fig. 5C. Strain BET22 produced 28.7 mg/L of betanin in baffled shake-flask, 79.6% higher than that in non-baffled one, as well as exhibited a significant biomass advantage. It indicated that increasing oxygen supply is beneficial for yeast growth and biosynthesis of betanin, consistent with previous report [3]. In addition, No accumulation of l-tyrosine and l-DOPA was detected in the baffled cultivation (Fig. S5C), while betalamic acid was slightly reduced (∼4%) compared to non-baffled cultivation (Fig. S5D). Finally, we evaluated the performance of strain BET22, under the optimal fermentation conditions. As shown in Fig. 5D, from 12 to 24 h, yeast consumed glucose rapidly and grew exponentially, while starting to produce betanin. After glucose depletion at 36 h, ethanol was used as carbon source to maintain cell growth and biosynthesis of betanin. After 72 h fermentation, betanin accumulated gradually to 28.7 mg/L with an average productivity of 9.6 mg/(L·day), which was higher than previous report of 7.1 mg/(L·day) with the titer of 14.2 mg/L in 48 h cultivation [3].

## 4 Conclusions

Overall, this study focused on the betanin biosynthetic pathway reconstitution and optimization in engineered *S. cerevisiae*, and a final betanin titer of 28.7 mg/L was obtained using glucose as the sole carbon source at 25 °C in baffled shake-flask fermentation for 72 h. To our knowledge, this is the highest reported titer of betanin produced by an engineered microorganism. CYP76AD1-α enzyme involves in biosynthesis of betanin, and its capacity might be improved by more subtle N-terminal modifications of truncation with or without fused reductase. Furthermore, the sufficient supply of precursor l-tyrosine has been developed in yeast [27,28], and we anticipate that future engineering efforts in enhancing the carbon flux toward l-tyrosine will achieve higher productivity of betanin.

## CRediT authorship contribution statement

**Lijuan Zhang:** Conceptualization, Investigation, Data curation, Formal analysis, Methodology, Visualization, Writing – original draft, Writing – review & editing, review & editing. **Xue Liu:** Investigation, Formal analysis, Methodology, Visualization, Writing – review & editing. **Jiawei Li:** Investigation, Data curation, Validation. **Yonghui Meng:** Investigation, Methodology. **Guang-Rong Zhao:** Conceptualization, Formal analysis, Funding acquisition, Project administration, Writing – review & editing.

## Declaration of competing interest

The authors declare that they have no known competing financial interests or personal relationships that could have appeared to influence the work reported in this paper.
