# Supplementary material for: Improvement of betanin biosynthesis in Saccharomyces cerevisiae by metabolic engineering
Source: Synth Syst Biotechnol. 2022 Nov 12;8(1):54–60. doi: 10.1016/j.synbio.2022.11.002 (PMC9672881; doi:10.1016/j.synbio.2022.11.002)
Supplement: Multimedia component 1 [file mmc1.docx]

**Supplementary Information for**

**Improvement of** **betanin biosynthesis in *Saccharomyces cerevisiae* by metabolic engineering**

Lijuan Zhang^a,b,1^, Xue Liu^a,b,1^, Jiawei Li^a,b^, Yonghui Meng^a,b^, Guang-Rong Zhao^a,b,^*

^a^ Frontiers Science Center for Synthetic Biology and Key Laboratory of Systems Bioengineering (Ministry of Education), School of Chemical Engineering and Technology, Tianjin University, Yaguan Road 135, Jinnan District, Tianjin 300350, China

^b^ Georgia Tech Shenzhen Institute, Tianjin University, Dashi 1^st^ Rd, Nanshan District, Shenzhen 518055, China

* Corresponding author. Frontiers Science Center for Synthetic Biology and Key Laboratory of Systems Bioengineering (Ministry of Education), School of Chemical Engineering and Technology, Tianjin University, Yaguan Road 135, Jinnan District, Tianjin 300350, China.

E-mail address: [grzhao@tju.edu.cn](mailto:grzhao@tju.edu.cn) (G.-R. Zhao).

**Table S1. Primers used in this study.**

| **Primers** | **Sequences (5’-3’)** |
| --- | --- |
| 1622b-left-F | AACATTTAAGTCACAAGGAGGAATAT |
| 1622b-left-R | AACTACTTTTCTTAAACTGTCAACAGC |
| 1622b-HIS3-F | TTGGCTGTTGACAGTTTAAGAAAAGTAGTTCTAGTACACTCTATATTTTTTTATGCCTC |
| TDH3p-HIS3-R | GTGGATGCCAGGAATAAACTGTCTACATAAGAACACCTTTGGTGG |
| TDH3p-F | ACAGTTTATTCCTGGCATCCA |
| Mj*c*DOPA5GT-TDH3p-R | ACCGTTAGTGTTCATCTTGATAGCAGTCATTTTGTTTGTTTATGTGTGTTTATTCGA |
| Mj*c*DOPA5GT-F | ATGACTGCTATCAAGATGAACAC |
| Mj*c*DOPA5GT-R | TTATTGCAGAGATGGTTCCAG |
| Mj*c*DOPA5GT-ENO2t-F | CAAGCTAAGCTGGAACCATCTCTGCAATAAAGTGCTTTTAACTAAGAATTATTAGT |
| ENO2t-R | AGGTATCATCTCCATCTCCCA |
| ENO2t-HXK1p-F | ATATGCATATGGGAGATGGAGATGATACCTTTTAGACGCTTTTAAGGAGTACG |
| HXK1p-R | CTTATTTTTTCAGTATTCTAATTGAGTTGTTTG |
| HXK1p-MjDODA-F | ACAACTCAATTAGAATACTGAAAAAATAAGATGAAAGGGACATACTATATTAATCACG |
| MjDODA-R | TCAGACGTCCGTTTTTTGAGTA |
| MjDODA-GPM1t-F | AACTTCACCCCTACTACTCAAAAAACGGACGTCTGAAGAATGAATGATTTGATGA |
| 1622b-GPM1t-R | TATATCCAATTTCGTAAGACGAGTATCTACTATTCGAACTGCCCATTCAGC |
| 1622b-right-F | GTAGATACTCGTCTTACGAAATTGGA |
| 1622b-right-R | ACTTTGGAAAAGAAGGTACGGAC |
| 1622b-yzF | ATGTCTCCTTATCCTTGAGTGC |
| 1622b-yzR | AAATTTGTTCCCTCAGATGTGAC |
| 308a-left-F | TATTTCAGAAAAATTATTCAAAACTAAGAAGAATGAG |
| 308a-left-R | TTAGATAAAAAGAAAAAAATTCGAAGTTAATGTTG |
| 308a-TRP1-F | TTAACTTCGAATTTTTTTCTTTTTATCTAAAACGACATTACTATATATATAATATAGGAAGCATT |
| TDH3p-TRP1-R | TTAGTGGATGCCAGGAATAAACTGTCTATTTCTTAGCATTTTTGACGAAATTTGCTAT |
| TDH3p-R | TTTGTTTGTTTATGTGTGTTTATTCG |
| TDH3p-BvAD1^W13L^-F | GAATAAACACACATAAACAAACAAAATGGACCACGCGACCCTG |
| GPDt-BvAD1^W13L^-R | ATTTAAATGCAAGATTTAAAGTAAATTCACTTAGTAGCGTGGGATAGGGAT |
| GPDt-F | GTGAATTTACTTTAAATCTTGCATTTAAATAAATTTTC |
| CCW12p-GPDt-R | GATTCCCAAAACGGAAATCAGACGCCAATAGGAATCTGTGTATATTACTGCATCTAGAT |
| CCW12p-F | TATTGGCGTCTGATTTCCGTT |
| CCW12p-R | TATTGATATAGTGTTTAAGCGAATGACAG |
| CCW12p-AtR1-F | TCTGTCATTCGCTTAAACACTATATCAATAATGACCAGCGCACTGTATGC |
| ADH1t-AtR1-R | TAATAAAAATCATAAATCATAAGAAATTCGCTTACCAAACATCACGCAGATAACG |
| ADH1t-F | GCGAATTTCTTATGATTTATGATTTTTATT |
| 308a-ADH1t-R | AATAGAAGTGGTAGCAATATGTAGCAAAGACGTAAAAAAAGCATGCACGTATAC |
| 308a-right-F | TCTTTGCTACATATTGCTACCACT |
| 308a-right-R | TGATAGAACGAGTACAACACCCG |
| 308a-yzF | CAGCGAAGATAACGGTTACAC |
| 308a-yzR | GACCGATTAAGCTATCCTTTTTG |
| TDH3p-CqAD1-F | GAATAAACACACATAAACAAACAAAATGGACCACGCTACTTTGG |
| GPDt-CqAD1-R | ATTTAAATGCAAGATTTAAAGTAAATTCACTTAGTATCTCAAGACTGGGATGATC |
| TDH3p-CcAD4-F | GAATAAACACACATAAACAAACAAAATGGACAACGCCACATTGGC |
| GPDt-CcAD4-R | ATTTAAATGCAAGATTTAAAGTAAATTCACTTAGTATCTGGAGACTGGGATGATTCT |
| TDH3p-AcAD1-F | GAATAAACACACATAAACAAACAAAATGGACAACGCTACTTTGGCT |
| GPDt-AcAD1-R | ATTTAAATGCAAGATTTAAAGTAAATTCACTTAACGAGATGGGATGATTTCCAA |
| TDH3p-HuAD1-F | GAATAAACACACATAAACAAACAAAATGGACTCTCCAACTTTGTG |
| GPDt-HuAD1-R | ATTTAAATGCAAGATTTAAAGTAAATTCACTTAGTCCTTACAAACTGGGATGAT |
| TDH3p-PaAD11-F | GAATAAACACACATAAACAAACAAAATGGACCACACTACTTTGG |
| GPDt-PaAD11-R | ATTTAAATGCAAGATTTAAAGTAAATTCACTTAGTACTTCAAGACTGGGATAACT |
| TDH3p-CbAD12-F | GAATAAACACACATAAACAAACAAAATGGACTACACTACCTTGGT |
| GPDt-CbAD12-R | ATTTAAATGCAAGATTTAAAGTAAATTCACTTAGTACTTGTAGACTGGGATGATT |
| TDH3p-BaAD14-F | GAATAAACACACATAAACAAACAAAATGGACAACACTACTTTGGC |
| GPDt-BaAD14-R | ATTTAAATGCAAGATTTAAAGTAAATTCACTTAGATAGAGTTCTTGAAGATTGGGAT |
| TDH3p-CctAD4-F | GTTTCGAATAAACACACATAAACAAACAAAATGTTGTTCACCAACCAATCCAC |
| CCW12p-AttR1-F | TCTGTCATTCGCTTAAACACTATATCAATAATGTGGAAAAAAACCACCGCAG |
| AtR1-CcAD4-R | GCGCTGGTACCAGATGAACCTGAAGAAGTAGAACCGTATCTGGAGACTGGGATGATTCT |
| CcAD4-AtR1-F | TCTCCAGATACGGTTCTACTTCTTCAGGTTCATCTGGTACCAGCGCACTGTATGCAAG |
| AttR1-CcAD4-R | TTTTTCCAACCAGATGAACCTGAAGAAGTAGAACCGTATCTGGAGACTGGGATGATTCT |
| CcAD4-AttR1-F | TCCAGATACGGTTCTACTTCTTCAGGTTCATCTGGTTGGAAAAAAACCACCGCAGATCG |
| CcAD4^W13L^-R | GGAGATGAACAAGATAGCCAACAA |
| CcAD4^W13L^-F | TTGTTGGCTATCTTGTTCATCTCC |
| delta1-F | AAAAATCCACTATCGTCTATCAACT |
| loxp-R | GAATTCGAGCTCGGTACCC |
| loxp-TDH3p-F | AAGTTATCCCGGGTACCGAGCTCGAATTCACAGTTTATTCCTGGCATCCA |
| CCW12p-Mj*c*DOPA5GT-F | TCTGTCATTCGCTTAAACACTATATCAATAATGACTGCTATCAAGATGAACAC |
| delta2-ENO2t-R | ATTAGGTATACAGAATATACTAGAAGTTCTCAGGTATCATCTCCATCTCCC |
| delta2-F | GAGAACTTCTAGTATATTCTGTATACCTAAT |
| delta2-R | AACAGCTGATGAAGCAGGTG |

**Table S2. The synthesized genes used in this study.**

| **Genes** | **Sequence (5’-3’)** |
| --- | --- |
| *MjcDOPA5GT* | ATGACTGCTATCAAGATGAACACTAACGGTGAAGGTGAAACTCAACACATCCTGATGATCCCATTCATGGCTCAAGGTCACCTGCGTCCATTCCTGGAACTGGCTATGTTCCTGTACAAGCGTTCTCACGTTATCATCACTCTGCTGACTACTCCACTGAACGCTGGTTTCCTGCGTCACCTGCTGCACCACCACTCTTACTCTTCTTCTGGTATCCGTATCGTTGAACTGCCATTCAACTCTACTAACCACGGTCTGCCACCAGGTATCGAAAACACTGACAAGCTGACTCTGCCACTGGTTGTTTCTCTGTTCCACTCTACTATCTCTCTGGACCCACACCTGCGTGACTACATCTCTCGTCACTTCTCTCCAGCTCGTCCACCACTGTGTGTTATCCACGACGTTTTCCTGGGTTGGGTTGACCAAGTTGCTAAGGACGTTGGTTCTACTGGTGTTGTTTTCACTACTGGTGGTGCTTACGGTACTTCTGCTTACGTTTCTATCTGGAACGACCTGCCACACCAAAACTACTCTGACGACCAAGAATTCCCACTGCCAGGTTTCCCAGAAAACCACAAGTTCCGTCGTTCTCAACTGCACCGTTTCCTGCGTTACGCTGACGGTTCTGACGACTGGTCTAAGTACTTCCAACCACAACTGCGTCAATCTATGAAGTCTTTCGGTTGGCTGTGTAACTCTGTTGAAGAAATCGAAACTCTGGGTTTCTCTATCCTGCGTAACTACACTAAGCTGCCAATCTGGGGTATCGGTCCACTGATCGCTTCTCCAGTTCAACACTCTTCTTCTGACAACAACTCTACTGGTGCTGAATTCGTTCAATGGCTGTCTCTGAAGGAACCAGACTCTGTTCTGTACATCTCTTTCGGTTCTCAAAACACTATCTCTCCAACTCAAATGATGGAACTGGCTGCTGGTCTGGAATCTTCTGAAAAGCCATTCCTGTGGGTTATCCGTGCTCCATTCGGTTTCGACATCAACGAAGAAATGCGTCCAGAATGGCTGCCAGAAGGTTTCGAAGAACGTATGAAGGTTAAGAAGCAAGGTAAGCTGGTTTACAAGCTGGGTCCACAACTGGAAATCCTGAACCACGAATCTATCGGTGGTTTCCTGACTCACTGTGGTTGGAACTCTATCCTGGAATCTCTGCGTGAAGGTGTTCCAATGCTGGGTTGGCCACTGGCTGCTGAACAAGCGTACAACCTGAAGTACCTGGAAGACGAAATGGGTGTTGCTGTTGAACTGGCTCGTGGTCTGGAAGGTGAAATCTCTAAGGAAAAGGTTAAGCGTATCGTTGAAATGATCCTGGAACGTAACGAAGGTTCTAAGGGTTGGGAAATGAAGAACCGTGCTGTTGAAATGGGTAAGAAGCTGAAGGACGCTGTTAACGAAGAAAAGGAACTGAAGGGTTCTTCTGTTAAGGCTATCGACGACTTCCTGGACGCTGTTATGCAAGCTAAGCTGGAACCATCTCTGCAATAA |
| *MjDODA* | ATGAAAGGGACATACTATATTAATCACGGCGACCCTCTGATGTACCTGAAAAAGCACATTAAATTGCGTCAGTTCCTGGAGGGTTGGCAGGAGAACGTTGTGATCGAAAAGCCGAAGTCCATTCTGATCATTTCCGCCCATTGGGACACTAACGTGCCGACTGTGAACTTCGTGGAACATTGCGACACCATCCACGATTTCGACGACTACCCGGACCCACTGTATCAGATCCAGTATCGCGCACCGGGTGCTCCTAACCTGGCTAAAAAAGTTGAGGAGCTGCTGAAAGAGTCTGGTATGGAGTGCGAAATTGATACCAAACGTGGTCTGGATCACGCAGCGTGGTTCCCGCTGATGTTCATGTATCCGGAAGCTAATATTCCTATTTGTGAACTCTCTGTTCAGCCGAGCAAAGACGGTATTCACCATTACAACGTGGGCAAGGCACTGTCACCGCTGCTCCAACAGGGTGTTCTGATCATCGGTTCGGGCGGCACCGTTCACCCGAGCGACGACACCCCGCACTGCCCGAACGGTGTTGCGCCCTGGGCTATCGAGTTTGATAACTGGCTGGAAGATGCCCTGCTGTCTGGTCGTTACGAAGATGTTAACAACTTTAAAAAGCTGGCACCGAACTGGGAAATCTCCCACCCGGGCCAGGAGCACCTGTATCCGCTGCACGTTGCACTGGGCGCGGCTGGTAAAAACCCGAAAACCCAACTGATTCATCGTTCCTGGGCGGCCAACGGCGTATTCGGTTATAGCACTTACAACTTCACCCCTACTACTCAAAAAACGGACGTCTGA |
| *AtR1* | ATGACCAGCGCACTGTATGCAAGCGATCTTTTTAAACAGCTGAAAAGCATTATGGGCACCGATAGCCTGAGTGATGATGTTGTTCTGGTTATTGCAACCACCAGTCTGGCACTGGTTGCAGGTTTTGTTGTGCTGCTGTGGAAAAAAACCACCGCAGATCGTAGCGGTGAACTGAAACCGCTGATGATTCCGAAAAGCCTGATGGCAAAAGATGAAGATGATGATCTGGATCTGGGTAGCGGTAAAACCCGTGTTAGCATCTTTTTTGGCACCCAGACCGGCACCGCAGAAGGTTTTGCCAAAGCACTGAGCGAAGAAATTAAAGCCCGTTATGAAAAAGCAGCCGTGAAAGTTATCGATCTGGATGATTATGCAGCAGATGACGATCAGTATGAAGAAAAACTGAAGAAAGAAACCCTGGCCTTTTTTTGTGTTGCAACCTATGGTGATGGTGAACCGACCGATAATGCAGCACGTTTTAGCAAATGGTTTACCGAAGAAAACGAGCGCGATATTAAACTGCAGCAGCTGGCCTATGGTGTTTTTGCACTGGGTAATCGCCAGTATGAACACTTTAACAAAATTGGCATTGTGCTGGATGAGGAACTGTGTAAAAAAGGTGCAAAACGTCTGATTGAAGTTGGTCTGGGCGACGATGATCAGAGCATCGAAGATGATTTTAATGCCTGGAAAGAAAGCCTGTGGTCAGAACTGGATAAACTGCTGAAAGATGAGGACGATAAAAGCGTTGCAACCCCGTATACCGCAGTTATCCCGGAATATCGTGTTGTTACCCATGATCCGCGTTTTACCACCCAGAAAAGCATGGAAAGCAATGTTGCAAATGGCAACACCACCATTGATATTCATCATCCGTGTCGTGTTGATGTTGCAGTTCAGAAAGAACTGCATACCCATGAAAGCGATCGTAGCTGTATTCATCTGGAATTTGATATTAGCCGTACCGGCATTACCTATGAAACCGGTGATCATGTTGGTGTGTATGCCGAAAATCATGTGGAAATTGTTGAAGAAGCAGGCAAACTGCTGGGTCATAGCCTGGACCTGGTTTTTAGCATTCATGCAGATAAAGAAGATGGTAGTCCGCTGGAAAGCGCAGTTCCGCCTCCGTTTCCGGGTCCGTGTACCCTGGGTACGGGTCTGGCACGTTATGCCGATCTGCTGAATCCGCCTCGTAAAAGCGCACTGGTGGCACTGGCAGCCTATGCAACCGAACCGAGCGAAGCAGAGAAACTGAAACATCTGACCAGTCCGGATGGTAAAGATGAATATAGCCAGTGGATTGTTGCAAGCCAGCGTAGCCTGCTGGAAGTTATGGCAGCATTTCCGAGCGCAAAACCGCCTCTGGGTGTTTTTTTTGCAGCAATTGCACCGCGTCTGCAGCCTCGTTATTATAGCATTAGCAGCTGTCAGGATTGGGCACCGTCACGTGTTCATGTTACCTCAGCACTGGTTTATGGTCCGACCCCGACAGGTCGTATTCATAAAGGTGTTTGTAGCACCTGGATGAAAAATGCAGTTCCGGCAGAAAAAAGCCATGAATGTAGTGGTGCACCGATTTTTATCCGTGCAAGCAACTTTAAACTGCCGAGCAATCCGAGCACCCCGATTGTTATGGTTGGTCCGGGTACAGGCCTGGCACCGTTTCGTGGTTTTCTGCAAGAACGTATGGCACTGAAAGAGGATGGCGAAGAACTGGGTAGCAGCCTGCTGTTTTTTGGTTGTCGTAATCGTCAGATGGATTTCATCTATGAGGATGAGCTGAACAACTTTGTTGATCAGGGTGTTATTAGCGAACTGATTATGGCATTTAGCCGTGAAGGTGCACAGAAAGAATATGTGCAGCATAAAATGATGGAAAAAGCCGCACAGGTTTGGGATCTGATTAAAGAAGAAGGTTATCTGTACGTTTGCGGTGATGCAAAAGGCATGGCACGTGATGTTCATCGTACCCTGCATACCATTGTTCAAGAACAAGAAGGTGTTAGCAGCAGCGAAGCCGAAGCAATTGTTAAAAAACTGCAGACCGAAGGTCGTTATCTGCGTGATGTTTGGTAA |
| *BvCYP76AD1^W13L^* | ATGGACCACGCGACCCTGGCGATGATTTTGGCTATTCTGTTCATTTCTTTCCACTTCATCAAACTGCTTTTCTCCCAACAGACCACTAAACTGCTGCCGCCGGGCCCGAAACCGCTGCCGATCATTGGCAACATCCTGGAAGTCGGCAAAAAACCGCACCGTTCCTTCGCTAACCTGGCCAAAATACACGGTCCGCTCATCAGCCTGCGTCTGGGCAGTGTGACCACCATCGTTGTGTCCAGCGCGGATGTGGCTAAAGAAATGTTCCTCAAAAAAGATCACCCGCTGTCTAACCGTACTATCCCTAACAGCGTCACTGCCGGTGATCATCACAAACTGACCATGTCTTGGCTGCCGGTGTCCCCGAAATGGCGCAACTTTCGCAAAATCACGGCCGTTCATCTGCTGAGCCCGCAGCGTCTGGATGCCTGCCAGACCTTCCGTCACGCGAAAGTTCAGCAACTGTACGAATACGTACAGGAGTGCGCGCAAAAAGGTCAGGCGGTAGACATTGGTAAGGCCGCGTTTACCACCTCCCTTAACCTACTTTCTAAATTATTCTTCTCTGTTGAGTTGGCGCACCATAAGTCGCACACCTCTCAGGAGTTTAAGGAATTAATCTGGAACATCATGGAAGACATTGGCAAACCCAACTACGCTGACTACTTTCCTATCTTAGGTTGCGTGGACCCGTCCGGTATCCGTCGTCGCCTGGCATGCAGTTTTGATAAACTGATCGCCGTATTCCAGGGCATCATTTGTGAACGTCTGGCACCTGACTCTTCTACCACAACCACCACTACCACTGACGATGTGCTGGATGTGCTGCTGCAACTGTTCAAACAGAACGAACTGACGATGGGTGAGATCAACCATCTGCTGGTTGACATTTTTGATGCGGGTACTGACACCACGTCCAGCACCTTTGAGTGGGTAATGACCGAGTTAATCCGCAACCCGGAAATGATGGAAAAAGCGCAGGAAGAAATCAAGCAAGTTCTGGGCAAAGACAAACAAATCCAAGAAAGTGATATTATCAATCTGCCGTATTTACAAGCGATTATCAAAGAAACCCTGCGTCTCCACCCACCGACCGTTTTCCTGCTGCCGCGTAAAGCGGACACAGATGTGGAACTGTACGGCTATATCGTGCCGAAAGATGCGCAGATCCTGGTCAATTTGTGGGCGATTGGTCGTGACCCGAACGCCTGGCAAAACGCGGACATCTTCTCGCCGGAGCGTTTCATCGGCTGCGAAATTGATGTGAAAGGTCGCGATTTTGGTCTGCTGCCGTTCGGTGCCGGTCGTCGCATTTGTCCGGGCATGAACCTGGCCATCCGCATGCTGACCCTGATGCTGGCCACCCTGTTACAATTCTTCAACTGGAAATTGGAGGGCGACATTAGCCCGAAAGACCTGGACATGGATGAAAAATTTGGTATCGCACTGCAAAAGACGAAACCGCTGAAATTAATCCCTATCCCACGCTACTAA |
| *CqCYP76AD1* | ATGGACCACGCTACTTTGGCTATGATCTTGGCTATCTGGTTCGTTGTTTTCCACTTCATCAAGATGTTGTTCACTTCTCAGACTACTAAGTTGTTGCCACCAGGTCCAAAGCCATTGCCATTGATCGGTAACATCTTGGAAGTTGGTGAAAAGCCACACCAGTCTTTCGCTAACTTGGCTAAGATCCACGGTCCATTGATCTCTTTGAGGTTGGGTTCTGTTACCACTATCGTTGTCTCTTCTGCTGAAGTTGCTAAGGAGATGTTCTTGAAGAAGGACCACCCATTGTCTAACAGAACTGTTCCAAACTCTGTTACTGCTGGTGACCACCACAAGTTGACTATGTCTTGGTTGCCAGTCTCTCCAAAGTGGAGAAACTTCAGAAAGATCACTGCTGTTCACTTGTTGTCTCCACAAAGATTGGACGCTTGTCAGACCTTGAGACACGCTAAGGTTCAACAATTGTTCCAATACGTTCAAGAGTGTGCTCAGAAGGGTCAAGCTGTTGACATCGGTAAGGCTGCTTTCACTACCTCTTTGAACTTGTTGTCTAAGTTGTTCTTCTCTGTTGAATTGGCTCACCACAAGTCTCACACTTCTCAACAGTTCAAGGAATTGATCTGGAACATCATGGAAGACATCGGTAAGCCAAACTACGCTGACTACTTCCCAATCTTGGGTTGCTTGGACCCATCTGGTATCAGAAGGAGATTGGCTTCTAACTTCGACAAGTTGATCGCTGTTTTCCAGTCTATCATCTGTCAAAGAATCGGTAACGGTCAAGACTCTGCTTCTACTAAGACTACTGACGACGTCTTGGACATCTTGTTGGACTTGCACAAGCAGAAGGAGTTGTCTATGGGTGAAATCAACCACTTGTTGGTTGACATCTTCGACGCTGGTACTGACACTACTTCTTCTACCTTCGAATGGGTTATGGCTGAATTGATCAGAAACCCAAAGATGATGGAGAAGGCTCAAGAAGAAATCGAACAAGTCTTGGGTAAGGACAGACAGATCCAAGAATCTGACATCATCAAGTTGCCATACTTGCAAGCTATCATCAAGGAAACCTTGAGATTGCACCCACCAACTGTTTTCTTGTTGCCAAGAAAGGCTGACTCTGACGTTGAGTTGTACGGTTACGTCGTTCCAAAGGACGCTCAAATCTTGGTTAACTTGTGGGCTATCGGTAGAGACCCACAAGCGTGGGTTAAGCCAGACGTTTTCTTGCCAGAAAGGTTCTTGGGTTCTGAAATCGACGTTAAGGGTAGAGACTTCGGTTTGTTGCCATTCGGTGCTGGTAGGAGAATCTGTCCAGGTATGAACTTGGCTATCAGAATGTTGACTTTGATGTTGGCTACTTTGTTGCAATTCTTCAACTGGAAGTTGGAGGAAGGTATGAAGGCTGAAGACTTGGACATGGACGAAAAGTTCGGTATCGCTTTGCAAAAGACCAAGCCATTGCAGATCATCCCAGTCTTGAGATACTAA |
| *CbCYP76AD12* | ATGGACTACACTACCTTGGTTATGATCTTGTCTATCGTTTTCTTCTGCTACAACTTGTTCAACTTGTTGTTCACTAGAAAGAACACTAAGTTGCCACCAGGTCCAAAGACTATCCCAATCTTCGGTAACATCTTCGAGTTGGGTAAGAAGCCACACCAGTCTTTCGCTAACTTGGCTAAGATCCACGGTCCATTGATGTCTTTGAAGTTGGGTTCTGTTACTACCATCGTTGTTTCTTCTGCTGAAGTTGCTAGAGAGATGTTCTTGAAGAACGACCAGTTGTTGTCTAACAGGACTGTTCCAAACTCTGTTACTGCTGGTGACCACCACAAGACCACTATGTCTTGGTTGCCAGTTTCTCAGAAGTGGAGAAACTTCAGGAAGATCACTGCTGTCCACTTGTTGTCTCCACAGAGATTGGACTCTTGCCAAGCTCTCAGACAAGCCAAGGTCAAGCAATTGTTCAACTACATCCACGAGTGTGCTCAGAAGGGTGAAGCTGTTGACATCGGTAAGGCTGCTTTCACTACCTCTTTGAACTTGTTGTCTAACTTGTTCTTCTCTGTCGAATTGGCTAACCACAAGTCTTCCTCTTCTCAAGAATTTAAGCAATTGATCTGGAACATCATGGAGGACATCGGTAAGCCAAACTACGCTGACTACTTCCCAGTCTTGAAGTACGTTGACCCATCTGGTATCAGAAGGAGATTGGCTTCTAACTTCAACAAGTTGATCGACGTCTTCCAAGGTTTCATCAGGTTGAGAATGTCTACTAACTCTTCTTGTGGTGCCACTAACCCAAACGACGTTTTGGACGTTTTGTTGAACTTGTACAAGGGTGACGACTTGAACATGGACGAGATCAACCACTTGTTGGTCGACATCTTCGACGCTGGTACTGACACTACTTCTTCTACTTTCGAGTGGGCTATGGCTGAATTGGTCAAGAACCCAAAGATGATGAAGAAGGCTCAAGCTGAGATCCAGCAAGTTTTGGGTAAGGACTCTATCATCAGAGAATCTGACATCCCAAACATGCCATACTTGCAAGCCATCATCAAGGAAACTTTGAGGTTGCACCCACCAACTGTCTTCTTGTTGCCAAGGAAGGCTGACGCTGACGTTGAGTTGTACGGTTACGTCGTTCCAAAGAACGCTCAAATCTTGGTTAACTTGTGGGCTTTGGGTAGAGACCCATTGGTTTGGAAGTCTCCAAACGTTTTCAAGCCAGAGAGATTCTTGGGTTCTGAAATCGACTTCAAGGGTAGAGACTTCGGTTTGTTGCCATTCGGTGCTGGTAGAAGGATCTGTCCAGGTATGAACTTGGCTTACAGAATGTTGACTTTGATGTTGGCTACTTTGTTGCAGTCTTTCGACTGGAAGGTTGCTGACGGTACTAACCCACAAGACATGGACATGGACGAAAAGTTCGGTATCGCTTTGCAGAAGACCACTCCATTGCAAATCATCCCAGTCTACAAGTACTAA |
| *CcCYP76AD4* | ATGGACAACGCCACATTGGCTATGTTGTTGGCTATCTGGTTCATCTCCTTCCACTTCATCAAGATGTTGTTCACCAACCAATCCACCAAGTTGTTGCCACCAGGTCCAAAACCATTGCCAATCATCGGTAACATCTTGGAAGTCGGTAAGAAGCCACACAGATCCTTCGCAAACTTGGCAAAGATCCACGGTCCATTGATCTCCTTGAAGTTGGGTTCCGTCACCACAATCGTCGTCTCTTCCGCAGAAGTTGCTAAGGAAATGTTCTTGAAGAAGGACCAACCATTGTCCAACAGAACCGTCCCAAACTCCGTTACTGCCGGTGATCATCATAAGTTGACCATGTCCTGGTTGCCAGTCTCTCCAAAATGGAGAAACTTCAGAAAGATCACCGCTGTCCACTTGTTGTCCCCATTGAGATTGGACGCTTGCCAATCTTTGAGACACGCTAAGGTCCAACAATTGTTCCAATACGTCCAAGAATGCGCTCAAAAGGGTCAAGCTGTCGACATTGGTAAGGCTGCTTTCACTACCTCCTTGAACTTGTTGTCCAAGTTGTTCTTCTCCAAGGAATTGGCTTCCCACAAGTCCAGAGAATCCCAAGAATTTAAGCAATTGATCTGGAACATCATGGAAGACATCGGTAAGCCAAACTACGCCGATTACTTCCCAATCTTGGGTTGCGTCGACCCATCTGGTATAAGAAGAAGATTGGCTTCCAACTTCGACAAGTTGATCGAAGTCTTCCAATGCATCATCAGACAAAGATTGGAAAGAAACCCATCCACCCCACCTACTAACGATGTTTTGGACGTCTTGTTGGAATTGTACAAGCAAAACGAATTGTCTATGGGTGAAATCAACCACTTGTTGGTCGACATCTTCGACGCTGGTACAGACACTACTTCCTCTACTTTCGAATGGGTCATGGCCGAATTGATCAGAAACCCAGAAATGATGGCTAAGGCTCAAGACGAAATCGAACAAGTCTTGGGTAAGGACAGACAAATCCAAGAATCCGACATCATCAAGTTGCCATACTTGCAAGCTATCATCAAGGAAACCTTGAGATTGCACCCACCAACCGTATTCTTGTTGCCAAGAAAGGCCGACACCGATGTAGAATTGTACGGTTACATCGTCCCAAAGGACGCTCAAATCTTGGTCAACTTGTGGGCTATCGGTAGAGACTCCCAAGCATGGGAAAACCCTAAGGTTTTCTCCCCAGACAGATTCTTGGGTTGCGAAATCGACGTCAAGGGTAGAGATTTCGGTTTGTTGCCATTCGGTGCCGGTAAAAGAATCTGCCCAGGTATGAACTTGGCTATCAGAATGTTGACCTTGATGTTGGCTACCTTGTTGCAATTCTTCAACTGGAAGTTGCAAGACGGTATGTCCTTGGAAGACTTGGACATGGAAGAAAAGTTCGGTATCGCTTTGCAAAAGACCAAGCCATTGAGAATCATCCCAGTCTCCAGATACTAA |
| *HuCYP76AD1-1* | ATGGACTCTCCAACTTTGTGGTTGTTCATCTTCGCTTCTATCTTCTACATCATCACCTTCCAGATCGTCAAGTTGGGTTTCAACGTTGTTATGACTTCTAAGAAGACTAAGAGAAGGAGACCACCATTGCCACCAGGTCCAAAGCCATTGCCAATCATCGGTAACGTTTTGGAATTGGGTCAGAAGCCACACAGATCGTTCGCTGACTTGGCTAAGGTTCACGGTCCATTGATGTCTTTGAGATTGGGTTCTGTTACTACCATCATCGTTTCTTCATCTGACGTTGCTAAGGAGATGTTCTTGAAGAACGACCAACCATTGTCTTCTTCTAGGACCATCCCAAACTCTGTTACTGCTGGTGACCACCACATGTTGACCATGTCTTGGTTGCCAGTTTCTCCAAAGTGGAGATCGTTCAGGAAGATCACTACCTTCCACTTGTTGTCTCCACAGAGGTTGGACGCTTGCTCTTCTTTGAGACAAGCTAAGGTTCAACAGTTGTTCGAGTACGTCTTGGAATGTTCTCGTACTGGTCAAGCTGTTGACATCGGTAAGGCTGCCTTCACTACCTCTTTGAACTTGTTGTCTAAGTTGTTCTTCTCTTTGGAATTGGCTCACCACAGATCGTCTAAGTGTCAAGAGTTCAAGGACTTGATCTGGGACATCATGGAAGACATCGGTAAGCCAAACTACGCTGACTACTTCCCATGCTTGAAGTACTTCGACCCATGTGGTATCAGAAGGAGATTGGCTAACTCTTTCGAAAAGTTGATCGAAGTCTTCCAAGGTATCATCAGACAAAGATTGTCTTTGTCTTCTGGTTCTCACACTCACAACGACGTCTTGGACGTTTTGTTGCAGTTGTACAACCAAGAAGAATTGACTATGGACGAAATCAACCACTTGTTGGTTGACATCTTCGACGCTGGTACTGACACTACCTCTTCTACCTTCGAGTGGGCTATGGCTGAATTGATCAAGAACAGAACTATGATGGAAAAGGCTCAAGCTGAAATCAAGGTTGTTTTGGGTAAGCAATCTCACATCCAGGAATCTGACATCCCAAAGTTGCCATACTTGAGAGCCATCATCAAGGAAACTTTGAGATTGCACCCACCAACTGTTTTCTTGTTGCCAAGAAAGGCTGAAACTGACGTTGAATTGTACGGTTACACTGTTCCAAAGAACGCTCAAATCTTGGTTAACTTGTGGGCTTTGGGTAGAGACCCAAAGGTTTGGGAGAACCCAGAAGTCTTCTTGCCAGAAAGATTCTTGACCTGTGACATCGACGTTAAGGGTAGAGACTTCGGTTTGTTGCCATTCGGTGCTGGTAGAAGGATCTGTCCAGGTATGAACTTGGCTTACAGAATGTTGACCTTGATGTTGGCTACTTTGTTGCAGTCTTTCGACTGGAAGTTGCCAAACGAAATGAACTCTAAGAACTTGGACATGGACGAGAAGTTCGGTATCGCTTTGCAGAAGACCAAGCCATTGGAAATCATCCCAGTTTGTAAGGACTAA |
| *AcCYP76AD1* | ATGGACAACGCTACTTTGGCTATGATCTTGACTATCTGGTTGATCTCTATCAACTTCATCAAGATGTTCTTCACTCACCAGAACACTAAGTTGATCTTGCCACCAGGTCCAAAGCCATTGCCAATCATCGGTAACATCTTGGAAGTTGGTAAGAAGCCACACAGGTCGTTCGCTAACTTGGCTAAGATCCACGGTCCATTGATCTCTTTGAGGTTGGGTTCTGTCACTACTATCGTTGTCTCTTCTGCTGAGGTTGCCAAGGAGATGTTCTTGAAGAAGGACCAGCCATTGTCTAACAGAAACGTTCCAAACTCTGTTACTGCTGGTGACCACCACAAGTTGACTATGTCTTGGTTGCCAGTCTCTCCAAAGTGGAGAAACTTCAGGAAGATCACTGCTGTTCACTTGTTGTCTCCATTGAGGTTGGACGCTTGCCAGTCTTTGAGACAAGCTAAGGTCCAACAGTTGTACCAATACGTTCAAGAATGCGCTCAGAAGGGTCAATCTATCGACATCGGTAAGGCTGCTTTCACTACTTCTTTGAACTTGTTGTCTAAGTTGTTCTTCTCTAAGGAGTTGGCTTGTCACAAGTCTCACGAGTCTCAAGAATTTAAGCAATTGATCTGGAACATCATGGAAGACATCGGTAAGCCAAACTACGCTGACTACTTCCCAATCTTGGGTTGCATCGACCCATTGGGTATCAGAAGAAGGTTGGCTGCCAACTTCGACAAGTTGATCGCTGTTTTCCAGACTATCATCTCTGAAAGGTTGGAAAACAACAAGAACGCTAACGCTACTAACGACGTTTTGGACGTCTTGTTGCAATTGTACAAGCAGAAGGAATTGTCTATGGGTGAAATCAACCACTTGTTGGTCGACATCTTCGACGCTGGTACTGACACTACTTCTTCTACTTTCGAATGGGTCATGACTGAATTGATCAGAAACCCAAACATGATGGAAAAGGCTCAACAAGAAATCCAAGAAGTTTTGGGTAAGGACAGACAGATCCAAGAATCTGACATCATCAAGTTGCCATACTTGCAAGCTCTCATCAAGGAAACTTTGAGATTGCACCCACCAACTGTCTTCTTGTTGCCAAGAAAGGCTGACATGGACGTCGAATTGTACGGTTACGTCGTTCCAAAGGACGCTCAAATCTTGGTTAACTTGTGGGCTATCGGTAGAGACCCACAAGCGTGGGAAAAGCCAAACGCTTTCTTGCCAGAAAGATTCTTGGGTTCTGACGTTGACGTCAAGGGTAGAGACTTCGGTTTGTTGCCATTCGGTGCTGGTAAGAGAATCTGTCCAGGTATGAACTTGGCTATCAGAATGTTGACTTTGATGTTGGCTACTTTGTTGCAGTTCTTCGACTGGAAGTTGGAAGAAGGTATGAACCCACAAGACTTGGACATGGACGAAAAGTTCGGTATCGCTTTGCAGAAGACTAAGCCATTGGAAATCATCCCATCTCGTTAA |
| *PaCYP76AD11* | ATGGACCACACTACTTTGGCTATGATCTTGTCTGTTATCTTCTTGTTGTACAACTTGGTCAAGGCTATCTTCTCTCAGTCTAACACTAAGTTGCCACCAGGTCCAAAGCCAGTTCCAATCTTCGGTAACATCTTCGAATTGGGTGACAAGCCACACAGATCGTTCGCTAACTTGGCTAAGATCCACGGTCCATTGATCACCTTGAAGTTGGGTTCTGTTACTACTATCGTTGTTTCTTCTGCTGAAGTCGCTAAGGAGATGTTCTTGACTAACGACCAGTTGTTGGCTAACAGGAACGTTCCAAACTCTGTCACTGCTGGTGACCACCACAAGTTGACCATGTCTTGGTTGCCAGTTTCTCCAAAGTGGAAGACTTTCAGAAAGATCACTGCTGTTCACTTGTTGTCTCCACAAAGGTTGGACGCTTGCCAAGCTCTCAGACACACTAAGGTCAAGCAGTTGTACGAGTACGTTCAAGAATGTGCTAAGAGAGGTGAAGCTGTTGACATCGGTAAGGCTGCTTTCACCACTTCTTTGAACTTGTTGTCTAACTTGTTCTTCTCTGTCGAATTGGCTAACCACACTTCTTCTTCTTCTCAAGAGTTTAAGGAATTGATCTGGGACATCATGGAAGACATCGGTAAGCCAAACTACGCTGACTACTTCCCAGTCTTGAAGTGTGTTGACCCCTGGGGTATCAGGAGAAGATTGGAGTCTAACTTCGACAAGTTGATCGAAGTTTTCCAGTCTTTCATCAGAAAGAGATTGTCTACTGAACCATTCTCTGCTTCTGCTAAGACTCCAAACGACGTTTTGGACGTTTTGTTGAACTTGTTGAAGGAAGAGGAATTGAACATGGGTGAAATCAACCACTTGTTGGTTGACATCTTCGACGCTGGTACTGACACTACCTCTTCTACTTTCGAATGGGCTATGGCTGAATTGGTTAGGAACCCAGAAATGATGAAGAAGGCTCAGGACGAGATCGAACAAGTTTTGGGTAAGGACGCTATCATCCAAGAATCTGACATCCCAAAGATGCCATACTTGCAAGCTATCATCAAGGAGACTTTGAGATTGCACCCACCAACTGTTTTCTTGTTGCCAAGAAAGGCTTCTTCTAACGTTGAGTTGTACGGTTACGTTGTTCCAAAGAACGCTCAAATCTTGGTTAACTTGTGGGCTATCGGTAGAGACCCAACTGTCTGGGACAACCCAAACATGTTCTCTCCAGAGAGGTTCTTGAACTCTGACATCGACGTTAAGGGTAGAGACTTCGGTTTGTTGCCATTCGGTGCTGGTAGGAGAATCTGTCCAGGTATGAACTTGGCTTACAGAATGTTGACTTTGATGTTGGCTACCTTGTTGCAGTCTTTCGACTGGAAGTTGGGTGACGGTGTTAACCCAAAGGACTTGGACATGGACGAGAAGTTCGGTATCGCTTTGCAAAAGACCAAGCCATTGCAAGTTATCCCAGTCTTGAAGTACTAA |
| *BaCYP76AD14* | ATGGACAACACTACTTTGGCTATCTTGTTGTCTACTTCTTACTTCATCATCTACTTGATCATCACCAAGTTGGGTTTCCACTACAAGGTTTTGCCAAAGAACCCAAAGCAGAGGTTGAGATTGCCACCAGGTCCAAAGCCATTGCCAATCATCGGTAACGTTTTGGAGTTGGGTTCTAAGCCACACAGGTCGTTCACCAACTTGGCTAAGGTTCACGGTCCATTGATCTCTTTGAGATTGGGTTCTGTTACTACCATCATCGTCTCTTCTTCTCACGTTGCTAAGGAAATGTTCTTGAAGAACGACCAGTCTTTGTCTTCTAACAGAACTATCCCACACTCTGTTACTGCTGGTGACCACCACAAGTTGACTATGTCTTGGTTGCCAGTTTCTCCAAAGTGGAGATCGTTCAGAAAGATCACTACCTTCCACTTGTTGTCTCCACAGAGGTTGGACGCTTGCTGCTCTTTGAGACAAGCTAAGGTTCAACAGTTGTTCGAGTACGTCTTGCAATGTTCTAGGACCGGTCAACCAGTCGACATCGGTAAGGCTGCTTTCACTACTTCTTTGAACTTGTTGTCTAAGTTGTTCTTCTCTTTGGAGTTGGCTCACCACAGGTCGACCAAGTCTCAAGAGTTTAAGGACTTGATCTGGAACATCATGGAAGACATCGGTAAGCCAAACATCGCTGACCACTTCCCATGCTTGAAGTACTTCGACCCATCTGGTATCAGAAGAAGATTGGCTTCTTCTTTCGAGAGATTGATCGAAGTTTTCCAAGACATCATCAGACAGAGGATGTCTTTGTCTTTCGGTTCTTCTCACAACAACGACGTTTTGGACGTTTTGTTGGGTTTGTACAACCAAAAGGAATTGACTATGGACGAAATCAACCACTTGTTGGTTGACATCTTCGACGCTGGTACTGACACCACTTCTTCTACTTTCGAATGGTCTATGGCTGAATTGATGAAGAACAGAAGAATCATGGAAAAGGCTCAAGCTGAAATCTTGCACGTTTTGGGTAAGAACTCTTACATCCAGGAATCTGACATCTCTAAGTTGCCATACTTGAGAGCTATCATCAAGGAAACTTTGAGATTGCACCCACCAACTGTCTTCTTGTTGCCAAGAAAGGCTGACGCTGACGTTGAGTTGTACGGTTACGTCGTTCCAAAGGACGCTCAAATCTTGGTTAACTTGTGGGCTTTGGGTAGAGACCCAGCTGTCTGGGAGAACCCAGACGAATTTTCTCCAGACAGGTTCATGGGTTCTGAGATCGACGTTAAGGGTAGAGACTTCGGTTTGTTGCCATTCGGTGCTGGTAGAAGGATCTGTCCAGGTATGAACTTGGCTATCAGAATGTTGACCTTGATGTTGGCTACTTTGTTGAGATCGTTCGACTGGAAGTTGCCAGAAGGTGAAGCTCCAGCTCAATTGGACATGGACGAAAAGTTCGGTATCGCTTTGCAGAAGACCACTCCATTGAAGATCATCCCAATCTTCAAGAACTCTATCTAA |

**Table S3. Chromosomal loci used in this study.**

| **Loci** | **Sequence (5’-3’)** |
| --- | --- |
| *1622b*-left | AACATTTAAGTCACAAGGAGGAATATCAGTTCTCTTCTCAATGGTAACAGAATCCATACTACCCTCTGCAATTGGTGGAAAAGCTGATGCCCTAATAATCAACAGTTTTTTTTCTGCCTGGCATTCTATTGTAGAAATAATGTTACCTGCATAAATTGGCCTTATAAAGGTCTTAGGATCTTTGATTACAGTAACCTCACAAACAGGTTGGACGTCCAAGAGCGCACCCACCCGAGGTAAAACACTTTTTCCAACAGAGGAGTTTGAGACAACAAAATGTGAATAGTCGCCGCCTTTTAATAGTTTCACTAATAACGGAGTTAGTTGTTCGGGAAGACAGGTATCTAATTTTGAATCTTCAAATATGACAAGCTTTTCTAAATTGCTGCATGAATATGAAGATTTTAGCGCCTCAGCAGTTTTTTCAGCTTTGCTACCTGTGATTACAGCTGTGATAGGGTTAGACAACTTTTGTGCAGCAGCCAATAAACTCAATGATGACCTTGAAACAGAGCCATCTTTTGAGCTTTCAATGAAAGCTAAAGTGGAGGCGTAATTTTTCTGGAGGAACTTTGCCTTGCTAGCTCTAGGCAAGACAGCAGCCAATGATTTAAACATAGAAATTAACAATTAGCTGCTTTACCGTTAATGGTAGTAAATTATATCACTAGATTTTGTTTGTTGATTATTTTAATTGTTTTTAATTTTTAGTTAGTAGATTTTCGTTCGAGGCCCTAAAAATGTACCGAAAAGTCATAATAAACGGGCGGAGTCACATCGTAAGAGTAACATATACAATATGCGAGCAGACTTTGTCCATTAAGCCAAGTAACAACAAAAGAACCAAAAAAAGGAAAGTATCTCGTCTCTGTTGAGTAATTCTTATATCTTGCAAGATTTAATTCTCACTACTAATACGGAACTCAATCAAAGTGGCTTAATCCCAAGACATGCATTGAATACGGTATCATTGGCTGTTGACAGTTTAAGAAAAGTAGTT |
| *1622b*-right | GTAGATACTCGTCTTACGAAATTGGATATAGTTACTCTCTAATGCTCAATCCATGTAAAGCATGAATCATCTCAACAGAAAAATCTGGGAGAGCATATTTGCAACAAAAGAATCCAAATATCAGTATATAATTATATAAGTGAGCTAAGAAGTGAACAAAAGAAAAGGAAGAGGGGAAAAATTTTGGCTGGACTTGTAGAACGATAGAACGAAAGAGTCCAAGGGGGAAGTAATAAAGTTATTGAACGAAACAACAAAAATTCGATGGAGATATTAACAAATGCATAGCAGCGCAGCTAACCGACCCGAAGCTAGTACCAACGAATTTTTCAACTATTCTGTGTTGATTGTCTCCTTTGCAACACCGAGCTCTTCAGTATTCTCAGCTGAAAGTTATTATTGAAATCACGTTGGGGACTTTCATGATCTAGTGGTCTTGGTGAGTAGCATTTAATATTTTGACCATTGAAAGGGCATCTCTCAATTGGAGAAAGGCCACTCTTAGGGATAGGCGTTTTAACAATCTGTTTATGGGATGAATGATATATTTTTGAGGATGGATTCACGATACTGATATGTTTCCCGTCCGCATTATTCGGATATGTAGTGCCTTCTTTCGATAAAATAGATGCCAATCCTGGCGTATGCTTTGTTTCGATGACGGAGCTTACCAATTTTTCACTTCGTATTTTCGATGAGTTCTTATTTTTACATTTGACGTTCAATATTCCGACATACGTCTCTCTTTCTGAAATGTTGGAACTCTGTGGCCTTGGATTTGGTGTTTTTTCACCCGAAAAATTGTTGATAACTGCACTTTTCTTTGAGGTACTACTATTTATATCTTCCTGCTCTTTTGTTCCCCGCTTAAGATGTTTTGGATGGCAAGTTTCAATAAACTGCGGTTTTATTCCTGCTTCTGAAGAGGTGCCCGAGATGTGAACCTCATTTCCACCACTACCAGTATAAGTAAAGTAGTCCGTACCTTCTTTTCCAAAGT |
| *308a*-left | TATTTCAGAAAAATTATTCAAAACTAAGAAGAATGAGATGATAAGAAAAACAGTTACCAATTTAGTAGCTGTAAGATTAAAAAACTTATCCCACGAATTTGATGTAATAGAGAATTATCTGCGCTATATAGCTAGCACCAGTGAACATCTATTTACTGCTATTAAGCGCCACTTTAACAAATGTGCCAGAAAACTTTTGAAAGAAGCAATCGACTCCAAATCAAACTCAGAAACTGCTACGGTGGTTCTGCAAGAAGGATTTTCTGGCATTTGCTTATTGAAAGCGTCTTCAATTATATTAAAACTAAAATTGAAGTTTCCAAAAAAGAAAGATAGAACTGATATTAGCAAATTGTGTGACAAGAAAGAACGGATGACACAGTGGTTAGAAATTTCAATTTTGATGAACTGAGAATAATAATATTATGCTCCCCTGGATTTTATGCGAAGACACTGCTGAAAAAATTTCTGATATATGCAGAGAAGGAGCAAATAAGCCAGTTTTAAGGAACCGAGATAAGCTGTTTTCGCCCATTGTTCTAGTTCTACAAAATATCTACAGGGAATCGATGAGGTTGTAAGAAATCCTGAATACTCTATGATAGTGCATAACACTAAAAAGTTGAAAGAATCCCGTATCATGGACGATTTCCTTGAACATTTGAGCAAAGACGATAACAAAGCATGGTATGGCGCGGAAGAAACCGAGAGAGCTGCAAAATTAGATGCAATAGAAACACTACTTATTACAGATAGTGTACTAAAAAGGAACGACGTGAAAAAACGTGAAAAATACCTAGACCTAATAGAGAATAGTGGAAACAACAATGGCAAAATATTCGTACTCAGTACTTCAAAAATCACAGTGAGCAACTTGACAAACCAACAGATATAGGCTGTGTCTTAAAATATACCATCCAATACCTTGATGAACTTTTCAAAGATGACTAAAATAAGTGAAATTTCAACATTAACTTCGAATTTTTTTCTTTTTATCTAA |
| *308a*-right | TCTTTGCTACATATTGCTACCACTTCTATTACACAATAGTTTCAATAGCTTGCAGCGTAGCTAAACTCTAAAATTTATCTAAATCACTCATATAAACCGAACCCTTCCCCTTCCGCTTATAGTACAGTACCTATACATTTCATAAACATGGCATGGCGATCAGCGCCAAACAATATGGAAAATCCACAGAAAGCTATTCATTGAAAAAATAGTACAAATAAGTCACATGATGATATTTGATTTTATTATATTTTTAAAAAAAGTAAAAAATAAAAAGTAGTTTATTTTTAAAAAATAAAATTTAAAATATTAGTGTATTTGATTTCCGAAAGTTAAAAAAGAAATAGTAAGAAATATATATTTCATTGAATGGATATATGAAACGTTTACTGGTGGAAGTTTTGCTCATATATTATTATTCAATAGAAGTAATAAAGAAAAAGTTGGTAAAGCAACTTAACAGTAAAAAGGTAATGATTGAAAAAGTTTTTGAACATCTAAGCTATATGTTGATGGGTTTACAATTTTACCATTAGTACTCATGCCTATACTTTTCTGTTCGTCCTTAATGTCCGCGATTTAGAGCAATCATTGAAAGTACTAGATACATTTTAGCCAGAGAGGACTCGTTGACGTAGAATTAAAATTCAAATGAATTTCCGCCCCATTCATATACCCCAAATAACAAACATATTAAAACTTCATAATTATTCAAAATGTGGAGTAGTATAGAAGAGCAGTACCTTCAAAATTGATTTCTTCAGTTTCCCACCCGGGATCCACTTGTCATGCGGTGAGAATCGTATATTGCGTATAATCCGTGTTTCATCACCCACATTATAGTACAAACCTACTGGTGTAACCATTATCATATTCATGACTTGTAGTTAAAAATCTTTCACGATAAATTGAGGAAAAAATCACGTTAAATAGTTTCATATCATTCTTTAGTTAAAGTCTGTGCATAAAAAGAGGATCGGGTGTTGTACTCGTTCTATCA |
| *delta1* | AAAAATCCACTATCGTCTATCAACTAATAGTTATATTATCAATATATTATCATATACGGTGTTAAGATGATGACATAAGTTATGAGAAGCTGTCATCGAAGTTAGAGGAAGCTGAAACGCAAGGATTGATAATGTAATAGGATCAATGAATATAAACATATAAAACGGAATGAGGAATAATCGTAATATTAGTATGTAGAAATATAGATTCCATTTTGAGGATTCCTATATCCTCGAG |
| *delta2* | GAGAACTTCTAGTATATTCTGTATACCTAATATTATAGCCTTTATCAACAATGGAATCCCAACAATTATCTCAACATTCACATATTTCTCATGGTAGCGCCTGTGCTTCGGTTACTTCTAAGGAAGTCCACACAAATCAAGATCCGTTAGACGTTTCAGCTTCCAAAACAGAAGAATGTGAGAAGGCTTCCACTAAGGCTAACTCTCAACAGACAACAACACCTGCTTCATCAGCTGTT |

**Table S4. CYP76AD1-α clade proteins discussed in this study.**

| **Protein** | **Protein ID** | **Identity (%)** | **Species** |
| --- | --- | --- | --- |
| CqCYP76AD1 | XP_021769302.1 | 88 | *Chenopodium quinoa* |
| CcCYP76AD4 | AGI78466.1 | 87 | *Celosia cristata* |
| AcCYP76AD1 | AKI33932.1 | 85 | *Amaranthus cruentus* |
| HuCYP76AD1-1 | HU03G00480.1* | 79 | *Hylocereus undatus* |
| PaCYP76AD11 | AJD87467.1 | 77 | *Phytolacca americana* |
| CbCYP76AD12 | AJD87468.1 | 76 | *Cleretum bellidiforme* |
| BaCYP76AD14 | AJD87470.1 | 74 | *Basella alba* |

Note: “*” indicates protein id from supplementary reference.

**
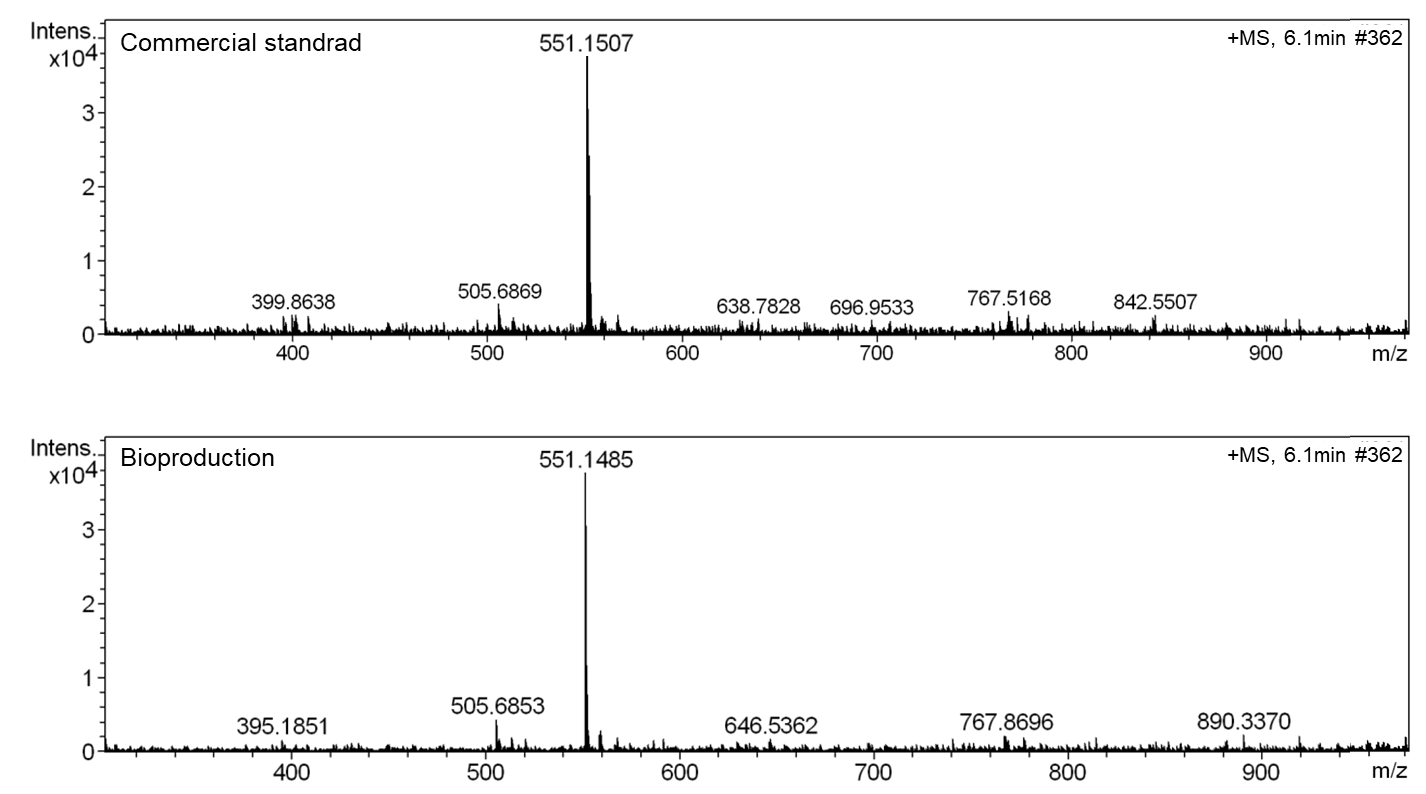
**

**Figure S1. LC-MS analysis for identification of betanin.**

**
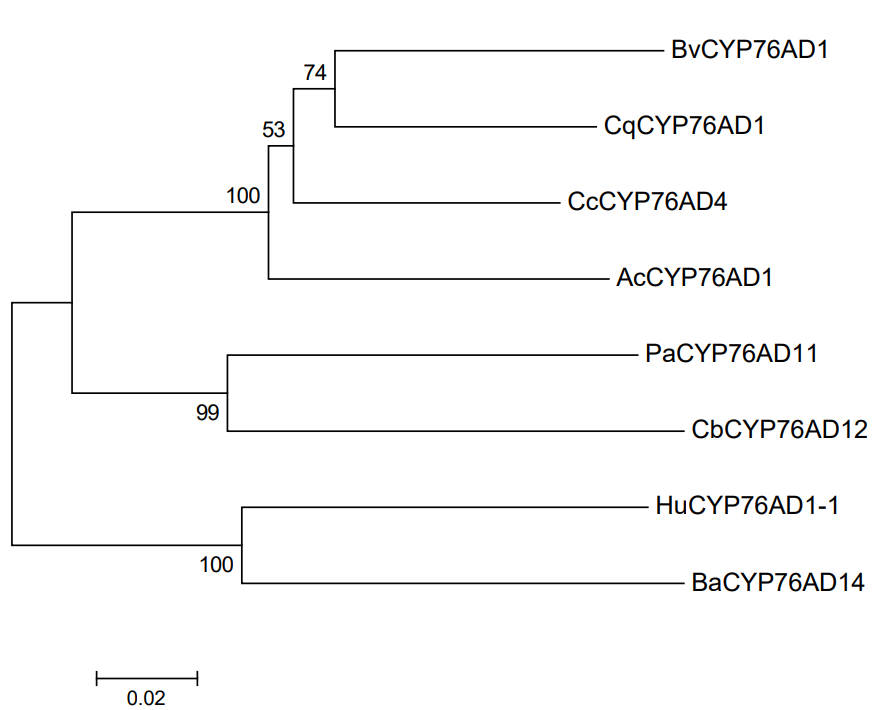
**

**Figure S2. Phylogenetic analysis of eight** **CYP76AD1-α clade proteins.** Neighbor-joining (NJ) tree was constructed using MEGA 7 with 1000 bootstrap replications.

BaCYP76AD14 MDNTTLAILL-STSYFIIYLIITKLGFHYKVLPK-NPKQRLRLPPGPKPLPIIGNVLELG 58

HuCYP76AD1-1 MDSPTLWLFIFASIFYIITFQIVKLGFNVVMTSKKTKRRRPPLPPGPKPLPIIGNVLELG 60

AcCYP76AD1 MDNATLAMILTI---WLIS-------INFIKMFFTHQNTKLILPPGPKPLPIIGNILEVG 50

BvCYP76AD1 MDHATLAMILAI---WFIS-------FHFIKLLFSQQTTK-LLPPGPKPLPIIGNILEVG 49

CqCYP76AD1 MDHATLAMILAI---WFVV-------FHFIKMLFTSQTTK-LLPPGPKPLPLIGNILEVG 49

CcCYP76AD4 MDNATLAMLLAI---WFIS-------FHFIKMLFTNQSTK-LLPPGPKPLPIIGNILEVG 49

PaCYP76AD11 MDHTTLAMILSV---IFLL-------YNLVKAIFSQSNTK--LPPGPKPVPIFGNIFELG 48

CbCYP76AD12 MDYTTLVMILSI---VFFC-------YNLFNLLFTRKNTK--LPPGPKTIPIFGNIFELG 48

** ** :: . : : ****** :*::**::*:*

BaCYP76AD14 SKPHRSFTNLAKVHGPLISLRLGSVTTIIVSSSHVAKEMFLKNDQSLSSNRTIPHSVTAG 118

HuCYP76AD1-1 QKPHRSFADLAKVHGPLMSLRLGSVTTIIVSSSDVAKEMFLKNDQPLSSSRTIPNSVTAG 120

AcCYP76AD1 KKPHRSFANLAKIHGPLISLRLGSVTTIVVSSAEVAKEMFLKKDQPLS-NRNVPNSVTAG 109

BvCYP76AD1 KKPHRSFANLAKIHGPLISLRLGSVTTIVVSSADVAKEMFLKKDHPLS-NRTIPNSVTAG 108

CqCYP76AD1 EKPHQSFANLAKIHGPLISLRLGSVTTIVVSSAEVAKEMFLKKDHPLS-NRTVPNSVTAG 108

CcCYP76AD4 KKPHRSFANLAKIHGPLISLKLGSVTTIVVSSAEVAKEMFLKKDQPLS-NRTVPNSVTAG 108

PaCYP76AD11 DKPHRSFANLAKIHGPLITLKLGSVTTIVVSSAEVAKEMFLTNDQLLA-NRNVPNSVTAG 107

CbCYP76AD12 KKPHQSFANLAKIHGPLMSLKLGSVTTIVVSSAEVAREMFLKNDQLLS-NRTVPNSVTAG 107

***:**:.***::***::*:*******:***:.**:****.:*: *: .*.:*:*****

BaCYP76AD14 DHHKLTMSWLPVSPKWRSFRKITTFHLLSPQRLDACCSLRQAKVQQLFEYVLQCSRTGQP 178

HuCYP76AD1-1 DHHMLTMSWLPVSPKWRSFRKITTFHLLSPQRLDACSSLRQAKVQQLFEYVLECSRTGQA 180

AcCYP76AD1 DHHKLTMSWLPVSPKWRNFRKITAVHLLSPLRLDACQSLRQAKVQQLYQYVQECAQKGQS 169

BvCYP76AD1 DHHKLTMSWLPVSPKWRNFRKITAVHLLSPQRLDACQTFRHAKVQQLYEYVQECAQKGQA 168

CqCYP76AD1 DHHKLTMSWLPVSPKWRNFRKITAVHLLSPQRLDACQTLRHAKVQQLFQYVQECAQKGQA 168

CcCYP76AD4 DHHKLTMSWLPVSPKWRNFRKITAVHLLSPLRLDACQSLRHAKVQQLFQYVQECAQKGQA 168

PaCYP76AD11 DHHKLTMSWLPVSPKWKTFRKITAVHLLSPQRLDACQALRHTKVKQLYEYVQECAKRGEA 167

CbCYP76AD12 DHHKTTMSWLPVSQKWRNFRKITAVHLLSPQRLDSCQALRQAKVKQLFNYIHECAQKGEA 167

*** ******** **:.*****:.***** ***:* :*::**:**::*: :*:: *:

BaCYP76AD14 VDIGKAAFTTSLNLLSKLFFSLELAHHRSTKSQEFKDLIWNIMEDIGKPNIADHFPCLKY 238

HuCYP76AD1-1 VDIGKAAFTTSLNLLSKLFFSLELAHHRSSKCQEFKDLIWDIMEDIGKPNYADYFPCLKY 240

AcCYP76AD1 IDIGKAAFTTSLNLLSKLFFSKELACHKSHESQEFKQLIWNIMEDIGKPNYADYFPILGC 229

BvCYP76AD1 VDIGKAAFTTSLNLLSKLFFSVELAHHKSHTSQEFKELIWNIMEDIGKPNYADYFPILGC 228

CqCYP76AD1 VDIGKAAFTTSLNLLSKLFFSVELAHHKSHTSQQFKELIWNIMEDIGKPNYADYFPILGC 228

CcCYP76AD4 VDIGKAAFTTSLNLLSKLFFSKELASHKSRESQEFKQLIWNIMEDIGKPNYADYFPILGC 228

PaCYP76AD11 VDIGKAAFTTSLNLLSNLFFSVELANHTSSSSQEFKELIWDIMEDIGKPNYADYFPVLKC 227

CbCYP76AD12 VDIGKAAFTTSLNLLSNLFFSVELANHKSSSSQEFKQLIWNIMEDIGKPNYADYFPVLKY 227

:***************:**** *** * * .*:**:***:********* **:** *

BaCYP76AD14 FDPSGIRRRLASSFERLIEVFQDIIRQRMSLSFGS---SHNNDVLDVLLGLYNQKELTMD 295

HuCYP76AD1-1 FDPCGIRRRLANSFEKLIEVFQGIIRQRLSLSSGS---HTHNDVLDVLLQLYNQEELTMD 297

AcCYP76AD1 IDPLGIRRRLAANFDKLIAVFQTIISERLENNKN---ANATNDVLDVLLQLYKQKELSMG 286

BvCYP76AD1 VDPSGIRRRLACSFDKLIAVFQGIICERLAPDSSTTTTTTTDDVLDVLLQLFKQNELTMG 288

CqCYP76AD1 LDPSGIRRRLASNFDKLIAVFQSIICQRIGNGQDSASTKTTDDVLDILLDLHKQKELSMG 288

CcCYP76AD4 VDPSGIRRRLASNFDKLIEVFQCIIRQRLERN---PSTPPTNDVLDVLLELYKQNELSMG 285

PaCYP76AD11 VDPWGIRRRLESNFDKLIEVFQSFIRKRLSTEPFSASAKTPNDVLDVLLNLLKEEELNMG 287

CbCYP76AD12 VDPSGIRRRLASNFNKLIDVFQGFIRLRMSTNS-SCGATNPNDVLDVLLNLYKGDDLNMD 286

.** ****** .*::** *** :* *: :****:** * : .:*.*

BaCYP76AD14 EINHLLVDIFDAGTDTTSSTFEWSMAELMKNRRIMEKAQAEILHVLGKNSYIQESDISKL 355

HuCYP76AD1-1 EINHLLVDIFDAGTDTTSSTFEWAMAELIKNRTMMEKAQAEIKVVLGKQSHIQESDIPKL 357

AcCYP76AD1 EINHLLVDIFDAGTDTTSSTFEWVMTELIRNPNMMEKAQQEIQEVLGKDRQIQESDIIKL 346

BvCYP76AD1 EINHLLVDIFDAGTDTTSSTFEWVMTELIRNPEMMEKAQEEIKQVLGKDKQIQESDIINL 348

CqCYP76AD1 EINHLLVDIFDAGTDTTSSTFEWVMAELIRNPKMMEKAQEEIEQVLGKDRQIQESDIIKL 348

CcCYP76AD4 EINHLLVDIFDAGTDTTSSTFEWVMAELIRNPEMMAKAQDEIEQVLGKDRQIQESDIIKL 345

PaCYP76AD11 EINHLLVDIFDAGTDTTSSTFEWAMAELVRNPEMMKKAQDEIEQVLGKDAIIQESDIPKM 347

CbCYP76AD12 EINHLLVDIFDAGTDTTSSTFEWAMAELVKNPKMMKKAQAEIQQVLGKDSIIRESDIPNM 346

*********************** *:**::* :* .** ** :***: *:**** ::

BaCYP76AD14 PYLRAIIKETLRLHPPTVFLLPRKADADVELYGYVVPKDAQILVNLWALGRDPAVWENPD 415

HuCYP76AD1-1 PYLRAIIKETLRLHPPTVFLLPRKAETDVELYGYTVPKNAQILVNLWALGRDPKVWENPE 417

AcCYP76AD1 PYLQALIKETLRLHPPTVFLLPRKADMDVELYGYVVPKDAQILVNLWAIGRDPQAWEKPN 406

BvCYP76AD1 PYLQAIIKETLRLHPPTVFLLPRKADTDVELYGYIVPKDAQILVNLWAIGRDPNAWQNAD 408

CqCYP76AD1 PYLQAIIKETLRLHPPTVFLLPRKADSDVELYGYVVPKDAQILVNLWAIGRDPQAWVKPD 408

CcCYP76AD4 PYLQAIIKETLRLHPPTVFLLPRKADTDVELYGYIVPKDAQILVNLWAIGRDSQAWENPK 405

PaCYP76AD11 PYLQAIIKETLRLHPPTVFLLPRKASSNVELYGYVVPKNAQILVNLWAIGRDPTVWDNPN 407

CbCYP76AD12 PYLQAIIKETLRLHPPTVFLLPRKADADVELYGYVVPKNAQILVNLWALGRDPLVWKSPN 406

***:*:*******************. :****** ***:*********:*** .* . .

BaCYP76AD14 EFSPDRFMGSEIDVKGRDFGLLPFGAGRRICPGMNLAIRMLTLMLATLLRSFDWKLPEGE 475

HuCYP76AD1-1 VFLPERFLTCDIDVKGRDFGLLPFGAGRRICPGMNLAYRMLTLMLATLLQSFDWKLPNEM 477

AcCYP76AD1 AFLPERFLGSDVDVKGRDFGLLPFGAGKRICPGMNLAIRMLTLMLATLLQFFDWKLEEGM 466

BvCYP76AD1 IFSPERFIGCEIDVKGRDFGLLPFGAGRRICPGMNLAIRMLTLMLATLLQFFNWKLEGDI 468

CqCYP76AD1 VFLPERFLGSEIDVKGRDFGLLPFGAGRRICPGMNLAIRMLTLMLATLLQFFNWKLEEGM 468

CcCYP76AD4 VFSPDRFLGCEIDVKGRDFGLLPFGAGKRICPGMNLAIRMLTLMLATLLQFFNWKLQDGM 465

PaCYP76AD11 MFSPERFLNSDIDVKGRDFGLLPFGAGRRICPGMNLAYRMLTLMLATLLQSFDWKLGDGV 467

CbCYP76AD12 VFKPERFLGSEIDFKGRDFGLLPFGAGRRICPGMNLAYRMLTLMLATLLQSFDWKVADGT 466

* *:**: .::*.***:*********:********* ***********: *:**:

BaCYP76AD14 APAQLDMDEKFGIALQKTTPLKIIPIFKNSI 506

HuCYP76AD1-1 NSKNLDMDEKFGIALQKTKPLEIIPVCKD*- 506

AcCYP76AD1 NPQDLDMDEKFGIALQKTKPLEIIPSR---- 493

BvCYP76AD1 SPKDLDMDEKFGIALQKTKPLKLIPIPRY-- 497

CqCYP76AD1 KAEDLDMDEKFGIALQKTKPLQIIPVLRY-- 497

CcCYP76AD4 SLEDLDMEEKFGIALQKTKPLRIIPVSRY-- 494

PaCYP76AD11 NPKDLDMDEKFGIALQKTKPLQVIPVLKY-- 496

CbCYP76AD12 NPQDMDMDEKFGIALQKTTPLQIIPVYKY-- 495

::**:**********.**.:**

**Figure S3. Multiple sequence alignment of CYP76AD1-α clade proteins.** The W13 residue is highlighted in yellow. Amino acids are color-coded based on their properties (red = small; blue = acidic; magenta = basic; green = hydroxyl, sulfhydryl, amine). “*” indicates fully conserved residues, “:” and “.” indicate strong and weak conservation respectively.

**
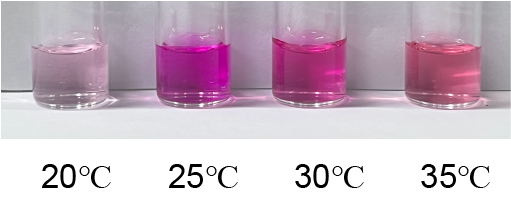
**

**Figure S4. Culture supernatants of strain BET22 cultivated at different temperatures.**

**
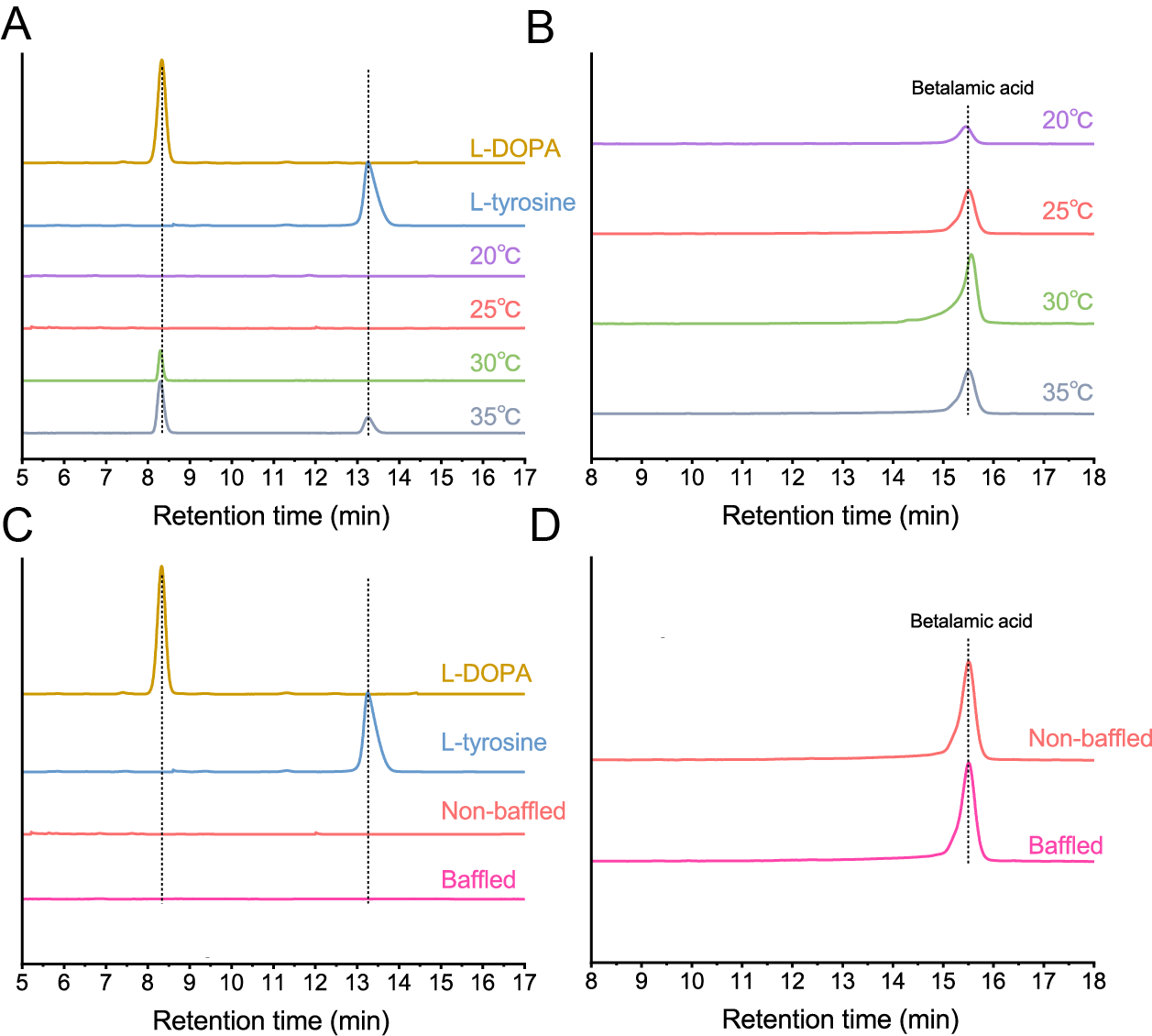
**

**Figure S5.** **The metabolic intermediates produced by strain BET22 under different fermentation conditions.** (A) HPLC spectra of L-tyrosine and L-DOPA at different temperatures. (B) HPLC spectra of betalamic acid at different temperatures. (C) HPLC spectra of L-tyrosine and L-DOPA in non-baffled and baffled shake-flask at 25°C. (D) HPLC spectra of betalamic acid in non-baffled and baffled shake-flask at 25°C.

**Supplementary Reference**

Chen J-Y, Xie F-F, Cui Y-Z, Chen C-B, Lu W-J, Hu X-D, et al. A chromosome-scale genome sequence of pitaya (*Hylocereus undatus*) provides novel insights into the genome evolution and regulation of betalain biosynthesis. Hortic Res 2021;8:164. <https://doi.org/10.1038/s41438-021-00612-0>.
